# Retracing Micro-Epidemics of Chagas Disease Using Epicenter Regression

**DOI:** 10.1371/journal.pcbi.1002146

**Published:** 2011-09-15

**Authors:** Michael Z. Levy, Dylan S. Small, Daril A. Vilhena, Natalie M. Bowman, Vivian Kawai, Juan G. Cornejo del Carpio, Eleazar Cordova-Benzaquen, Robert H. Gilman, Caryn Bern, Joshua B. Plotkin

**Affiliations:** 1 Center for Clinical Epidemiology & Biostatistics, Department of Biostatistics & Epidemiology, University of Pennsylvania School of Medicine, Philadelphia, Pennsylvania, United States of America; 2 Department of Statistics, Wharton School, University of Pennsylvania, Philadelphia, Pennsylvania, United States of America; 3 Department of Biology, University of Pennsylvania, Philadelphia, Pennsylvania, United States of America; 4 Johns Hopkins Hospital, Johns Hopkins University, Baltimore, Maryland, United States of America; 5 Asociacion Benefica PRISMA, Lima, Peru; 6 Gerencia Regional del Ministerio de Salud, Cercado Arequipa, Perú; 7 Departamento de Microbiología, de la Facultad de Medicina de la Universidad Nacional de San Agustín, Arequipa, Perú; 8 Bloomberg School of Public Health, Johns Hopkins University, Baltimore, Maryland, United States of America; 9 Division of Parasitic Diseases, Centers for Disease Control and Prevention, Atlanta, Georgia, United States of America; University of Texas at Austin, United States of America

## Abstract

Vector-borne transmission of Chagas disease has become an urban problem in the city of Arequipa, Peru, yet the debilitating symptoms that can occur in the chronic stage of the disease are rarely seen in hospitals in the city. The lack of obvious clinical disease in Arequipa has led to speculation that the local strain of the etiologic agent, *Trypanosoma cruzi*, has low chronic pathogenicity. The long asymptomatic period of Chagas disease leads us to an alternative hypothesis for the absence of clinical cases in Arequipa: transmission in the city may be so recent that most infected individuals have yet to progress to late stage disease. Here we describe a new method, epicenter regression, that allows us to infer the spatial and temporal history of disease transmission from a snapshot of a population's infection status. We show that in a community of Arequipa, transmission of *T. cruzi* by the insect vector *Triatoma infestans* occurred as a series of focal micro-epidemics, the oldest of which began only around 20 years ago. These micro-epidemics infected nearly 5% of the community before transmission of the parasite was disrupted through insecticide application in 2004. Most extant human infections in our study community arose over a brief period of time immediately prior to vector control. According to our findings, the symptoms of chronic Chagas disease are expected to be absent, even if the strain is pathogenic in the chronic phase of disease, given the long asymptomatic period of the disease and short history of intense transmission. Traducción al español disponible en Alternative Language Text S1/A Spanish translation of this article is available in Alternative Language Text S1

## Author Summary

Chagas disease has become an urban problem in the city of Arequipa, Peru, yet there are very few people exhibiting severe symptoms of the disease. Severe symptoms often do not appear until decades after infection. To determine why so few people were exhibiting severe symptoms, we used a new method, epicenter regression, to trace the history of Chagas disease transmission in a community of Arequipa, Peru. Our findings suggest that transmission in Arequipa occurred through a series of small epidemics, the oldest of which began only around 20 years ago. These micro-epidemics infected nearly 5% of the community before the insect that carries Chagas disease, *Triatoma infestans*, was eliminated by insecticide application. Most human infections in the study community arose over a brief period of time immediately prior to the insecticide application. According to our findings, the severe symptoms of Chagas disease are expected to be absent from the community because of the short duration of infection with the parasite, even among older individuals with Chagas disease.

## Introduction

Chagas disease, responsible for more deaths in the Americas than any other parasitic disease [1], has become an urban problem in the city of Arequipa, Peru [2], [3]. Nevertheless the debilitating symptoms of chronic Chagas disease, common across southern South America, are rarely seen in hospitals in the city (see Supp. Info Text S1). The lack of obvious disease in hospitals and the general population in Arequipa has led to speculation among local physicians that the local strain of the etiologic agent, *Trypanosoma cruzi,* has low chronic pathogenicity (personal observation), even though it has caused fatal acute infections in infants [4] and animal models[5], [6].

The vast majority of the 8–10 million individuals infected with *T. cruzi* [7] have the indeterminate form of Chagas disease. These individuals exhibit no symptoms or signs of their infection, but 20% to 30% are expected to progress to cardiac or digestive forms of chronic Chagas disease, which are difficult to treat and potentially fatal [8]–[10]. Progression to clinically evident cardiac disease is a slow process[8], [10]. For vector-borne transmission, decades may pass from the time of initial infection through contact with the feces of an infected triatomine bug until the onset of cardiac symptoms. Treatment with existing antitrypanosomal drugs, benznidazole or nifurtimox, appears to slow or prevent disease progression [9], but treatment is thought to be more effective when administered early in the course of infection [11].

The long asymptomatic period of Chagas disease leads us to an alternative hypothesis for the absence of clinical cases in Arequipa: transmission in the city may be so recent that most infected individuals have yet to progress to late stage disease. In order to evaluate this hypothesis it is necessary to elucidate the timing of *T. cruzi* infection in the population. The date of infection with *T. cruzi* is rarely known for individuals with Chagas disease. No existing assay of parasite or host factors yields reliable clues to the duration of infection. The dynamic process of *T. cruzi* transmission, however, creates clear spatial and temporal patterns of infection in insect vectors [12]-[14] and human hosts [15]–[20].

Traditionally, analyses of infectious diseases have aimed either to describe risk factors for infection at a static moment in transmission, using statistical methods to smooth heterogeneities in exposure between individuals created by the agent's spread [21]–[22], or to model the agent's spread through time and space, assuming a homogenous population [23]. Recent work has taken a more unified approach, estimating parameters of spread and local risk factors together [24]–[25]. Outside of a handful notable exceptions, especially [26]-[27], most unified analysis of disease spread and local risk factors address situations in which the time and place of the introduction of the disease agent is known. Here we develop a regression model, ‘epicenter regression,’ to infer the temporal and spatial spread of a disease agent for the more common situation in which the site or sites of introduction of a disease agent is unknown.

*Trypanosoma cruzi* is a slow-moving parasite. When it is transmitted in an epidemic phase, the period of time during which each individual is exposed to infection varies greatly based on how far away he or she lives from the site of introduction of the parasite. The heterogeneity in exposure time that results from the slow spread of the parasite may result in observable spatial clustering of infections. Observed spatial clustering, however, may also arise in endemic transmission if local risk factors for infection are concentrated in certain areas. Epicenter regression explicitly models the duration of an individual's exposure as a function of the distance of their household to an (unknown) site of introduction of a disease, then, given the exposure time of each individual's household, estimates the effect of risk factors measured in the house on the probability of infection for each individual. We fit models to patterns of *T. cruzi* infection in insect vectors and humans hosts in the peri-urban community of Guadalupe, Arequipa, Peru [3], [15]. We use Bayesian methods and Monte Carlo Markov Chains (MCMCs), an approach which allows us to make inference on parameters that reflect uncertainty about unknown factors, such as the location of cases in a dynamic infectious process [26]–[28].

We consider models with a single site of introduction of *T. cruzi* as well as models with multiple sites and times of introductions leading to multiple micro-epidemics. By ‘micro-epidemic’ we mean a focus of transmission, seeded from the same introduction of the parasite, that is discrete and discernable from the larger pattern of transfomission in the community. We also compare these models to an endemic model, in which each individual is assumed to have been exposed to the parasite since birth. Under the endemic model clustering of infection is explained mainly by clustering of household risk factors. Using the estimates from these models, we calculate the expected prevalence of infection among the population for each calendar year up to disruption of transmission through insecticide application in 2004. We discuss how a more precise understanding of the history of transmission might explain the absence of late-stage Chagas disease in Arequipa, and potentially inform clinical management of individuals with indeterminate Chagas disease.

## Methods

### Cross-sectional community survey

We conducted cross-sectional entomologic and serologic surveys in one recent settlement (*pueblo joven*), Guadalupe, on the southwestern margin of the city of Arequipa, Peru [14]. Vector-borne transmission of Chagas disease in Guadalupe was disrupted through application of deltamethrin insecticide in November of 2004. Concurrent to insecticide application, vector presence and density were determined through one person-hour timed search by trained professionals. Live and moribund fifth instar and adult triatomines were examined for *T. cruzi* consecutively for each site until 1 positive insect was found, 10 negative insects had been examined, or all available insects had been examined, whichever came first [14]. In August of 2005, all residents of the community were invited to participate in a serological survey for Chagas disease [15]. The positions of all households in Guadalupe were determined with a handheld global positioning system (Garmin Corporation, Olathe, KS, USA). Sera were screened using a commercial enzyme-linked immunosorbent assay (ELISA) kit with an epimastigote lysate antigen (Chagatek, Biomerieux, Buenos Aires, Argentina) following the manufacturer's instructions. Positive ELISAs were confirmed by an immunofluorescent antibody assay (IFA) following standard methods; a titer of 1∶32 was considered positive. Statistical analysis of data from Guadalupe was approved by the Institutional Review Board of the University of Pennsylvania.

### Epicenter regression model

Epicenter regression makes inference on where and when a disease agent was introduced into a community, as well as the effect of household-level covariates on the risk of infection given exposure. We begin with a simple model [29] in which an individual has a constant risk of being infected once exposed. The probability that an individual is infected is equal to 1 minus the probability of the individual escaping infection: *1 - e ^-Risk given exposure^*  ^*Duration of exposure*^. This equation is known as the catalytic model, because it also describes the probability of a change in state of molecules exposed to a constant bombardment of a catalyst [30]–[31].

We expand this framework into a biologically plausible model by allowing ‘risk given exposure’ and ‘duration of exposure’ to vary among individuals depending on observed covariates, and estimate the effect of the covariates from the observed data. We estimate the risk of exposed individual, *i*, due to the covariates measured in their household, *j*, using a traditional method to estimate risk, a log-linear model: *Risk_i_ =* 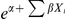. Here, *X_i_* represents a vector of covariates measured in each individual, and the *β* parameter describes how those covariates increase or decrease the log of the risk to each individual living in the exposed household. We assume that the effect of each covariate is constant, and varies neither year-to-year nor location-to-location. The intercept term, *α*, denotes the baseline risk of exposed individuals when all covariates are zero. We examined covariates previously shown to be associated with *T. cruzi* infection in children in the community. These included the presence of animals, almost exclusively dogs and cats, sleeping in the domestic area of the household and the number of vectors in the domestic portion of the household during timed entomologic search [15].

For each individual we estimated the period of time over which he or she had been exposed to *T. cruzi* as the lesser of two more-readily observable or estimable correlates of exposure: the individual's age, and the duration of exposure of their household. We assume the time of exposure of a household is a function of three unobserved parameters: *T,* the length of time since the introduction of *T. cruzi* in the community; *d*, the distance of the household from the household into which the parasite was first introduced**,** and, *r*, the rate of spread of the parasite. In the present analysis, we assume that a household becomes exposed at a time proportional to its distance from the site of introduction of the parasite. This assumption is a common simplification of mathematical analysis of invasive organisms based on diffusion equations, which concludes that, when individuals are assumed to move according to random walks, the wave front of the population will advance at a constant rate away from the site of introduction [23]. The time after the introduction *until* the parasite reaches the household, during which the residents of the household are not exposed, is: *d_jk_/r*, and the total duration of exposure is *T- d_jk_/r*, where *d_jk_* is the distance of household *j* from the location of introduction *k*. The probability of infection of individual *i* under the model is: 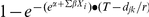 for all individuals born before their household was exposed to vectors carrying the parasite; and: 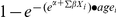 for all individuals born after their household was exposed to vectors carrying the parasite.

A steep hilltop separated households in the study area. We calculated the distance between the households going around this hilltop. As in the related proportional hazards model [32], time of exposure in epicenter regression must be limited to positive values. In the above model if *d/r* is greater than *T*, then time of exposure is set to zero, and the probability of infection is equal to zero. We used data on the presence of *T. cruzi* in insects to further refine our estimates of parameters *T*, *d* and *r*. For parameter regimes in which the model predicted no exposure for households in which we had observed *T. cruzi* in vectors, we set the likelihood to zero.

### Multiple introductions

We expanded our epicenter regression framework to consider the possibility that *T. cruzi* was introduced into Guadalupe on multiple occasions. Each introduction would occur in a different household, *k,* at a different time, *T_k_*. We calculate the duration of exposure of individuals in household *j* due to introduction into household *k* as: *T_k_ – (d_jk_/r)*. We assume that once transmission is established in a household it remains established. We also assume that the introduction of additional parasites into a household in which transmission is already established does not incrementally increase the risk of infection among those living in the house. The duration of exposure of each individual is therefore the maximum of their exposure to each introduction, or their age if their household was exposed prior to their birth. The probability of infection of each individual is otherwise as calculated above.

### Endemic model

For comparison purposes, we fit an endemic model, in which we assume that each individual's time of exposure was equivalent to their age. The infection probability of individual *i* living in household *j* under the endemic model is: 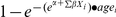.

### Incorporation of prior knowledge

Bayesian analysis of epicenter regression begins with what is known about the epidemic before testing people in the community, the “priors” of the model [33]. A key assumption of the model is that the parasite was introduced into a household and is spreading from the site of introduction. Before observing the data we have no information about into *which* household (or households) the parasite was first introduced, and therefore set a uniform prior distribution on the probability that each household was a site of introduction. We set non-informative prior distrifbutions (Gaussian with a mean of zero and a standard deviation of 10^3^) on the effect of household-level covariates (the *βs*). The community of Guadalupe was 40 years old at the time of data collection. We therefore set a uniform prior, bounded at 1 and 40 years, on the time since introduction of the parasite, for each introduction, *T_k_*.

Providing an explicit prior on the speed of spread of the pathogen allows us to better tailor our analysis to the particulars of *T. cruzi* transmission by *T. infestans.* We based our prior on the speed of spread of the wavefront of *T. cruzi* on longitudinal data from Villa la Joya, a community similar to Guadalupe in terms of household density, history, and animal husbandry practices. In Villa la Joya we surveyed 30% of households in January of 2008, uncovering 6 sites of *T. cruzi* infection among triatomine bugs. We conducted an exhaustive survey in conjunction with insecticide application by the ministry of health, following the methodology used in Guadalupe, in November and December of 2008. We identified forty-four additional foci of *T. cruzi* in vectors, in well-defined clusters around pre-existing sites of parasite presence, during the exhaustive survey. Assuming that the parasite had spread from the center of each cluster, it would have traveled approximately 18 meters over the course of 10–12 month interval between the preliminary and exhaustive surveys in order to reach each of the 44 foci. In no case had the parasite spread farther than 58 meters from a pre-existing site. Based on these findings, we assigned a normal distribution, with a mean of 20 meters and a variance of 100 meters, to describe the prior probability on the yearly speed of the spread of the parasite in Guadalupe. A spread rate of 20 meters/year is akin to a single parasitic household infecting on average between 6 and 7 additional households a year when introduced into a susceptible community.

### Model fitting, comparison, and prediction

We fit epicenter regression models using Bayesian methods and Monte Carlo Markov Chains (MCMCs). We updated MCMCs using the Metropolis and Metropolis-Hastings algorithms [33] (see annotated code in technical appendix: Text S3, Dataset S1, Text S4, Text S5). For the endemic model and models with 1 to 10 introductions, we ran 50 replicate MCMC chains, each of a length of 1 million estimates. We discarded the first 100,000 and retained every 10^th^ estimate in the remainder of a chain to diminish autocorrelation among the estimates. For each pair of models compared, we estimated the Bayes factor by the average, over the 50 pairs of chains, of the ratio of harmonic means of the posterior likelihood for the models [34]. We assured convergence of the chains using Geweke's test [35] as well as the Gelman-Rubin statistic [36]. We considered models with 1 through 10 introductions of parasite into the population (see movies in supplemental materials: Text S2, Video S1, Video S2, Video S3, Video S4). We estimated the prevalence for each calendar year under alternative models by integrating the risk of infection per unit of time over the time period that each individual was exposed. Finally, we quantified the expected number of cases of late-stage Chagas disease among individuals infected with *T. cruzi* at the time of the study. The time between infection and development of late stage disease is note fully known, but generally estimated at between 10 and 30 years [8]. We used three alternative models to describe the probability of onset of late-stage disease as a function of time: 1. A Poisson distribution centered at 20 years; 2. A Gaussian distribution with a mean of 20 years and 95% of the probability density between 10 and 30 years; and, 3. A uniform distribution bounded at 10 and 30 years. For each of these we integrated over the predicted temporal dynamics and calculated the expected number of cases, and the probability of observing exactly zero cases. We used a conservatively high estimate of the proportion of individuals who will eventually develop late stage disease, 30% [8].

## Results

**Figure 1:**
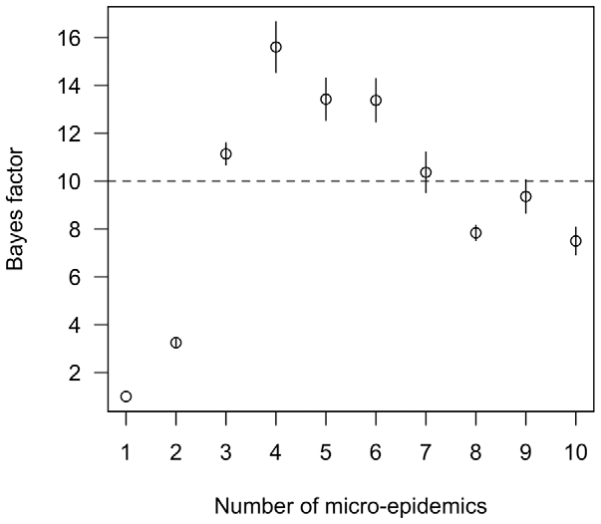
Comparison of the fit of multi-epidemic models for describing *T. cruzi* transmission in Guadalupe, Arequipa, Peru to a single epidemic model. Shown are the mean and standard error of the estimated Bayes' factors for comparing each model to the 1 epicenter model. The dotted line denotes models with strong support relative to the 1 epicenter model (Bayes' factor >10).

Models describing *T. cruzi* transmission as epidemic fit the data collected in the community of Guadalupe much better than an alternative, endemic model. The odds in favor of the epidemic models compared to the endemic model increased more than 4 orders of magnitude after considering the observed data – i.e. the Bayes' factors comparing the epidemic models to the endemic model exceeded 10^4^ (Figure 1). A Bayes' factor of greater than 10 is considered ‘strong evidence’ in favor of one model over another [34]. Models describing transmission of *T. cruzi* as a series of focal micro-epidemics were better supported than a model with a single epidemic stemming from one site of introduction. In particular, a model with four micro-epidemics had the greatest support from the observed data; the odds in favor of this model compared to the single-epidemic model increased 15-fold after considering the observed data (Figure 1).

**Figure 2:**
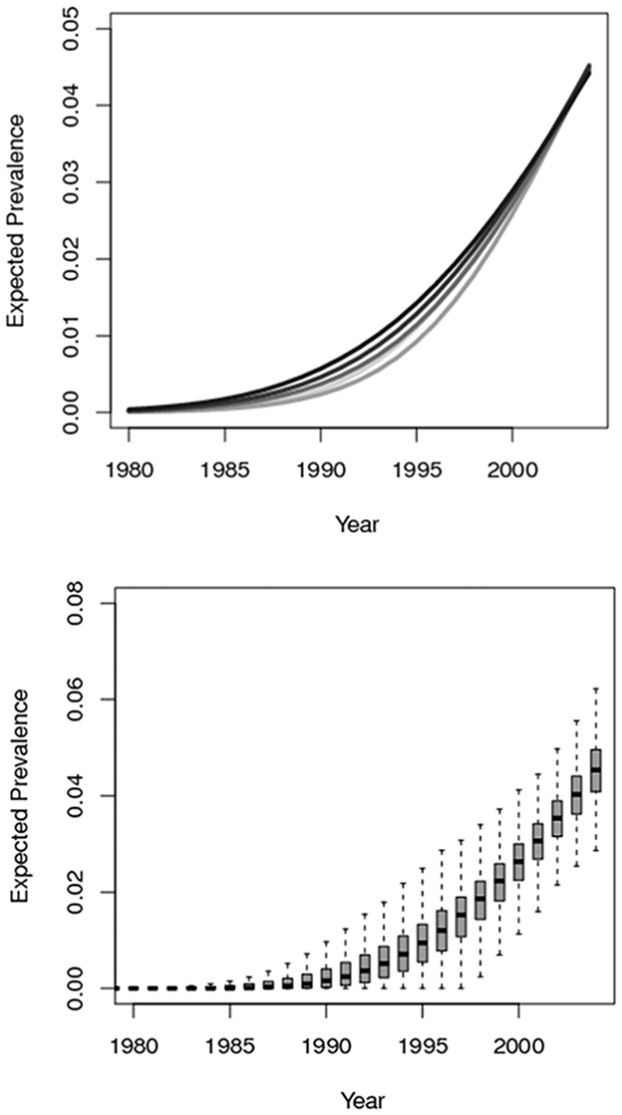
The expected percent of study participants infected over each calendar year back to 1980. A. The expectation for models with 2,4,6,8 and 10 epicenters; lines are shaded according to the number of epicenters (2 epicenters-light grey, 10 epicenters = black). B. A boxplot showing the median and credible intervals for the posterior estimates from the best-fit four-epicenter regression model. Chagas disease is a lifelong infection; infected individuals are assumed to remain seropositive through their lifetimes.

Under the four-epicenter model, the parasite was first introduced into Guadalupe about 20 years ago. When we tabulated the exposure time and risk of infection of individuals in the population in the four-epicenter model we found that around half of infections occurred in the 5 years previous to disruption of transmission through insecticide application, and 90% of infections occurred over a period of 12 years (Figure 2). These estimates were consistent across models with 2 to 10 epicenters (Figure 2). In contrast, under the endemic model, prevalence increased slowly over a much longer time frame.

**Figure 3:**
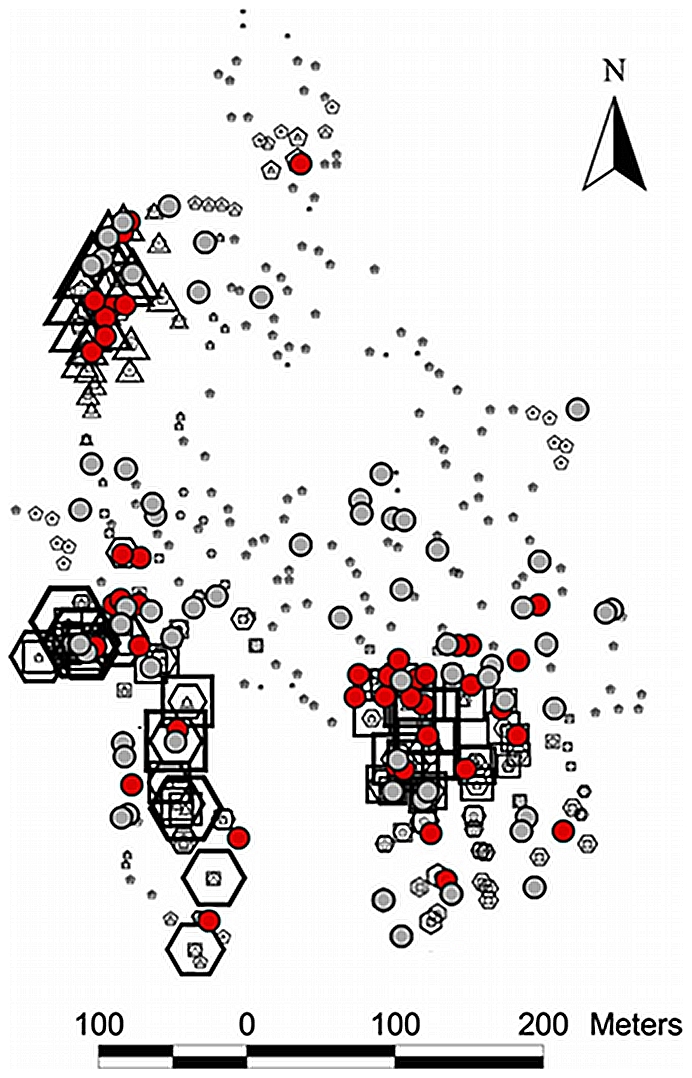
Estimated geographic position of introductions of *Trypanosoma cruzi* into the community of Guadalupe, Arequipa, Peru. The probability that each household was the first (hexagons), second (triangles), third (squares) or fourth (pentagons) site of introduction under the four-epicenter model is shown. Larger shapes correspond to higher probabilities. Households with cases of human disease are shown as red circles, and those with vectors carrying *T. cruzi* are shown in grey circles. The following steps were taken to protect patient anonymity in making this map: The geographic position of human cases has been slightly and randomly perturbed; the positions of some uninfected households have been altered; and certain regions of the map have been rotated a random angle around their centroid.

Spatially, the first introduction in the four-epicenter model occurred in the southwest of the community (Figure 3). A second micro-epidemic was then seeded in the northwest of the community, followed by a third in the southeast. Estimates on the position of the fourth micro-epidemic varied; some centered on a household with vectors carrying *T. cruzi* to the far east of Guadalupe, while others were nearer to a household with a human case in the far north of the community. Models with more than four introductions generally further subdivided these micro-epidemics; such divisions decreased the statistical support for these models.

**Figure 4:**
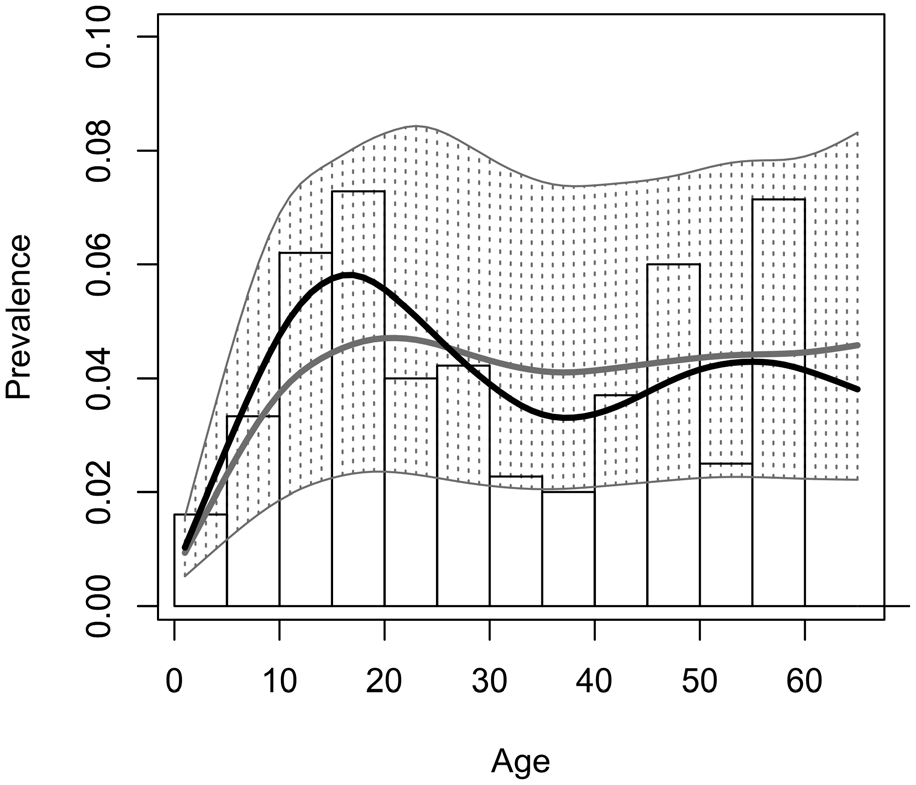
The observed and predicted relationship between age and prevalence of *Trypanosoma cruzi* infection in Guadalupe, Arequipa, Peru. The histogram represents the observed data; a smoothed spline, weighted by the number of observations at each age, is fit to these data (black curve). Model estimates of the relationship between age and prevalence were calculated by determining the probability of infection for each individual derived from the posterior predictions of the epicenter regression model with four epicenters. The spline fit to the median posterior predictions is surrounded by a region bounded by splines fit to predictions from the 2.5% and 97.5% quantiles of the posterior (light grey, shaded).

Temporally the four-epicenter model captured the relationship between age and prevalence observed in the data (Figure 4). Estimates of the time of the first introduction of *T. cruzi* into the community were remarkably similar across epidemic models (19.98 years ago in the one-epicenter model, 20.31in the four-epicenter model). The estimated effect of covariates on the risk of infection given exposure were also similar, with a 1–2% increase of risk per bug caught in the domestic area of the household and a 40–60% increase in risk among exposed individuals who allowed animals to sleep inside the domestic area of the household at night (Table 1). Posterior estimates on the rate of spread of *T. cruzi*, however, varied greatly. In the best-fit four-epicenter model a single exposed household would expose on average 4–5 additional households per year in a susceptible community. By contrast, in the one-epicenter model, the estimated rate of spread would expose an unrealistically large number of households (on average 15 households/year in a susceptible community). The higher estimated spread rate was counterbalanced by a lower estimated risk of infection given exposure in the one-epicenter model compared to the four-epicenter model.

**Table 1:** Posterior estimates and credible intervals for endemic, single-epidemic and four micro-epidemic models of *Trypanosoma cruzi* transmission in peri-urban Arequipa.

Parameter	Endemic Model	Single epidemic model	Four micro-epidemic model
	Median [2.5%, 97.5% cri 1]	Median [2.5%, 97.5% cri]	Median [2.5%, 97.5% cri]
Rate of Spread of the Parasite (m/year)	-	29.18 [17.52–44.69]^2^	17.35 [8.87–32.55]^3^
Baseline yearly risk of infection	0.0014 [0.0009–0.0020]	0.0032 [0.0018–0.0058]	0.0042 [0.0022–0.0086]
Relative risk per domiciliary insect captured	1.018 [1.004–1.028]	1.014 [1.000–1.024]	1.014[1.000–1.025]
Relative risk in households with animals sleeping inside	1.34 [0.66–2.56]	1.53 [0.76–2.93]	1.42 [0.70–2.72]
Years since the first introduction of the parasite	-	20.31 [12.71–33.25]	19.98 [10.92– 34.65]

1 Credible intervals are the 2.5^th^ and 97.5^th^ quantiles of the posterior samples.

2 Corresponds to an average of 15.01 [5.07, 34.26] households exposed by a single infected household in a fully-susceptible population.

3 Corresponds to an average of 4.950 [1.01, 18.77] households exposed by a single infected household in a fully-susceptible population.

**Figure 5:**
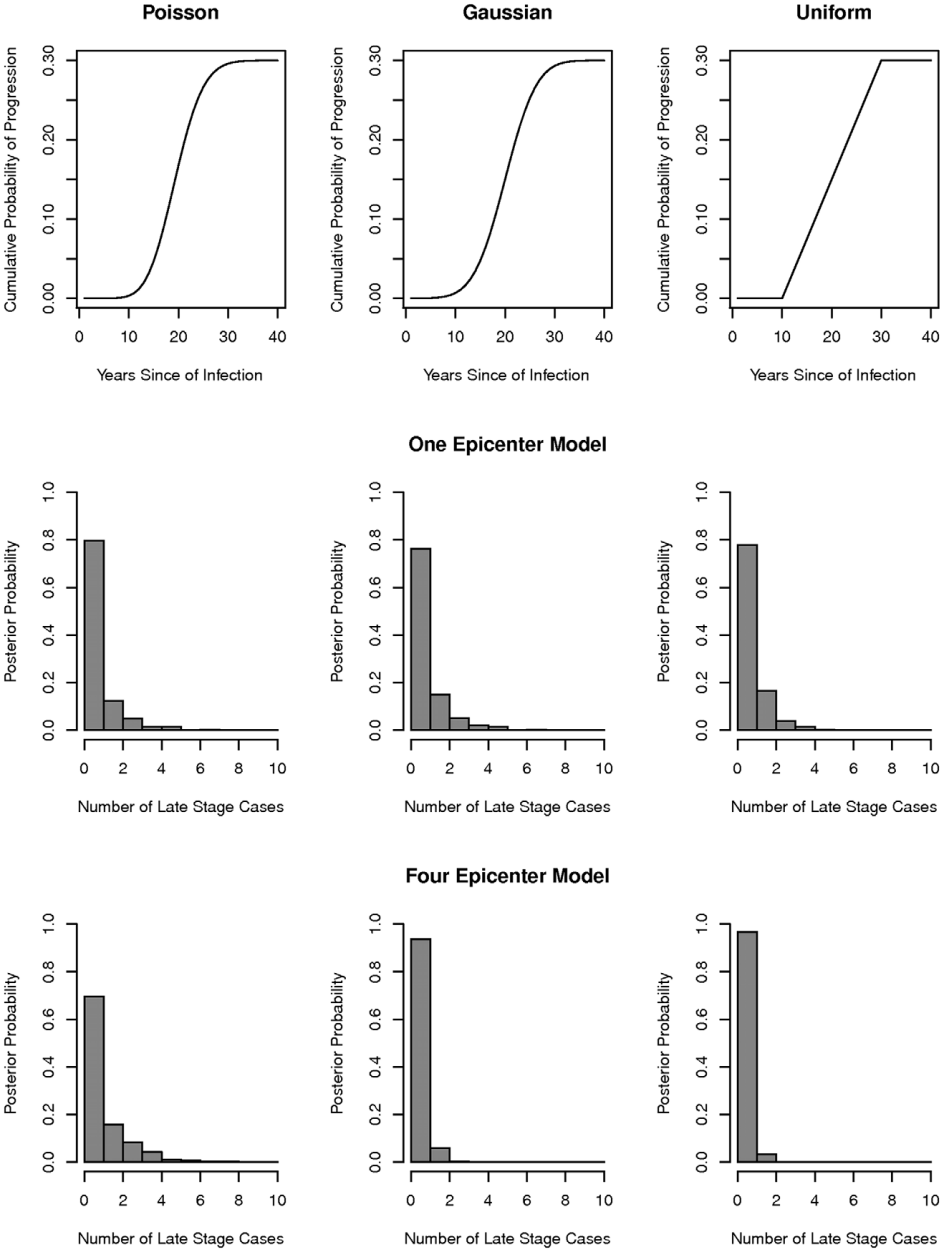
The expected number of cases of late-stage Chagas disease among individuals infected with *T. cruzi* in Guadalupe, Arequipa, Peru in 2004. Top row: three alternative models to describe the probability of onset of late-stage disease as a function of time (see text). Histograms represent the posterior expected number of late stage cases under the four-epicenter model (middle row) and one-epicenter model (bottom row).

When we combined our posterior model estimates of the timing of infection with *T. cruzi* with three alternative models of the distribution of waiting times between infection with the parasite and development of late stage Chagas disease, we found very high probabilities of observing less than one case of late stage disease in Guadalupe at the time of our survey (Figure 5). Under the four-epicenter model the probabilities of observing less than one case of late-stage disease were 0.696, 0.937, 0.967 under the Poisson, Gaussian and uniform models respectively. Given the posterior estimates of the timing of *T. cruzi* infection under the one-epicenter model, the probabilities of observing fewer than one case were 0.797, 0.763, 0.779 under the Poisson, Gaussian and uniform models.

## Discussion

Transmission of *Trypanosoma cruzi* in peri-urban Arequipa occurs in a series of spatially-focal micro-epidemics. The oldest of these micro-epidemics in the community of Guadalupe began only around 20 years ago. By the time vector-borne transmission of *T. cruzi* was disrupted through insecticide application in 2004, prevalence of human infection had reached 5% and was rapidly climbing. The relatively high prevalence of infection seems to conflict with the paucity of patients with Chagas cardiomyopathy in local hospitals, leading some to conclude that the strain of parasite in the region has low chronic pathogenicity. However, we have shown that most infections in Guadalupe likely occurred over a brief period of time prior to insecticide application. Our results provide support for a different explanation for the lack of late-stage Chagas disease in the city: the damage done by the vector and parasite may be unobserved because most individuals have yet to pass from the long asymptomatic period to symptomatic Chagas disease.

Our findings do not disprove the hypothesis that the parasites circulating in Arequipa are less pathogenic than other strains. Previous studies suggest that *T. cruzi* strains in Arequipa are of limited genetic diversity, possibly due to a founder effect [37]–[38]—a finding, which if true, would be consistent with the results presented here. We have typed fifteen isolates of *T. cruzi* from Arequipa following methods described in [39]. The majority of strains, though not all, are *T. cruzi* type I, including isolates from communities neighboring Guadalupe [*Vitaliano Cama*, *personal communication*]. It is absolutely possible that the particular strains of *T. cruzi* I that predominate in Arequipa cause less chronic pathology. However, we emphasize that, based on the results from our models, the lack of late-stage Chagas cardiomyopathy in peri-urban Arequipa cannot be taken as evidence of a weaker parasite, and that in the absence of such evidence, preparations should be made for an increasing burden of clinical disease in the region over the coming years.

Clinically, our findings may contribute to a re-evaluation of treatment guidelines for indeterminate Chagas disease that is currently underway [40]–[41]. Drug treatment is generally thought to be more effective early in the course of *T. cruzi* infection [11]. Currently many countries, Peru included, do not routinely offer treatment to patients over 15 years of age [1]. One justification for this policy is based on the assumption that age is a good surrogate for exposure to the parasite, and a reasonable surrogate of duration of infection among cases. That is, in the absence of evidence to the contrary, older individuals are assumed to have been carrying the parasite for many years, and therefore to be less likely to benefit from drug treatment. This assumption may be correct when transmission follows an endemic pattern. However, when transmission occurs in an epidemic, or multiple micro-epidemics, the assumption is incorrect. In peri-urban Arequipa and other epidemic situations, age alone is not a valid surrogate for exposure. Temporal and spatial information taken together give a better picture of how long an individual has been exposed to *T. cruzi*.

Geographically, our finding that *T. cruzi* was first introduced in the southwest of Guadalupe makes sense. Guadalupe is a peninsula, surrounded on three sides by fields that provide no habitat to triatomine vectors, while the southwest of the community borders a large hillside of similar settlements with documented Chagas disease transmission [3]. Once *T. cruzi* was introduced into the southwest of Guadalupe it is clear that it did not spread house to house through a simple diffusion process. Instead the parasite was transported to other sites in the community, and there seeded new micro-epidemics of transmission. We can only guess at the mechanism of transport of the parasite. Animals, especially guinea pigs, are commonly gifted or traded both within and between communities; infected guinea pigs could have sparked new micro-epidemics in Guadalupe. Infected insects can also fly to establish new foci of transmission; while the chance that any one insect establishes transmission is very small [42], given a large number of dispersing insects, establishment might occur occasionally. Individual-level data on human migration histories might allow us to study whether any infected individual was likely to have brought the parasite to Guadalupe from elsewhere [43]. Our finding of a very recent history of infections was robust to the precise number of micro-epidemics, and it is unlikely to be affected if these micro-epidemics were initiated through one mechanism rather than another.

Generally, our approach is applicable to any situation in which the expected observation of an organism at sampling locations at a certain time is a function of the (unknown) site or sites of introduction of the organism into the system. The functional relationship between the expectation at a sampling site to the site(s) of introduction can be a simple function of distance, as we have used here, or can include information about the habitat between the sampling and introduction sites. The method can be informative when some prior information, on the advance of the disease agent or the likely sites of introduction, is available. The method is not likely to be informative in the absence of both.

Our application of epicenter regression to *T. cruzi* transmission simplifies what is, in reality, a complex transmission cycle, and it is limited by the cross-sectional nature of the data. Our prior information on the speed of spread of the parasite came from empirical observations from another part of Arequipa; nevertheless the inherently stochastic nature of biological dispersal brings into question our ability to extrapolate our observations in one community to another [44]. Measurement of the Bayes' factor is also limited by errors of estimation [34]. Our primary goal was to estimate the timing of *T. cruzi* infections, as opposed to estimating the precise number of introductions of the parasite in the community. Although the four micro-epidemic model was strongly supported over the single-epicenter model, and substantially supported over the two-epicenter model, there was little difference in fit among models with 3-10 micro-epidemics. Other methods, such as reversible jump MCMC [45], might be more efficient when the number of introductions is of primary interest [28].

Our study focused on a single peri-urban community. Since the completion of our study we have observed similar patterns of micro-epidemics of *T. cruzi* transmission in entomologic data across the southern half of the city of Arequipa (unpublished data). In more rural areas outside the city, *T. cruzi* transmission was disrupted in the mid 1990s [46]. There are additional anecdotal reports of a lack of late-stage disease among individuals with Chagas disease residing outside of the city. A lack of late-stage disease among individuals infected many decades ago might provide evidence of lower chronic pathogenicity. In contrast, a finding of higher prevalence of late-stage disease among such individuals would provide direct evidence against this hypothesis.

The traditional, endemic patterns of transmission of *Trypanosoma cruzi* by *Triatoma infestans* have been largely disrupted across southern South America through a concerted vector control program known as the Southern Cone Initiative [1]. Currently the initiative is challenged by vectors and parasites returning to areas previously under insecticide control [13], [40]–[42], and by new foci of transmission in and around urban centers [14], [15]. The micro-epidemics of Chagas disease transmission we observed in Arequipa may be typical following emergence or re-emergence of the vector and parasite, rather than an anomalous pattern. Distinguishing between epidemic and endemic transmission will improve understanding of the dynamic relationship between prevalence of *T. cruzi* infection and the burden of clinical Chagas disease.

## Supporting Information

Alternative Language Text S1 **Spanish translation of the article by Denisse Barbu Covantes.**
(PDF)

Dataset S1 **Simulated Data for Epicenter Regression.** Simulated data from a Susceptible Infectious (SI) model on a grid to be used when running the epicenter regression and movie codes.
(CSV)

Text S1 **Evidence of Lack of Late Stage Chagas Disease.**
(DOCX)

Text S2 **Description of epicenter regression variables in video.** A visual compendium to the animations of describing the fitting process of the Monte Carlo Markov Chains for epicenter regression.
(PDF)

Text S3 **Read Me Code Explanation.** A ‘Readme’ file describing the accompanying epicenter regression codes.
(DOCX)

Text S4 **Epicenter Regression Model Code.** Epicenter Regression code (in R). The code is annotated throughout to describe the Monte Carlo Markov Chain fitting process.
(R) Click here for additional data file.

Text S5 **Epicenter Regression Movie Code.** R code used to produce movies similar to those in the supplemental materials of the manuscript.
(R) Click here for additional data file.

Video S1 **One -Epicenter Animation.** 1 Epicenter Model: Animation describing the Monte Carlo Markov Chain fitting process of epicenter regression to *T. cruzi* infection data in Arepuipa, Peru.
(MP4)

Video S2 **Two-Epicenter Animation.** 2 Epicenter Model: Animation describing the Monte Carlo Markov Chain fitting process of epicenter regression to *T. cruzi* infection data in Arepuipa, Peru.
(MP4)

Video S3 **Three-Epicenter Animation.** 3 Epicenter Model: Animation describing the Monte Carlo Markov Chain fitting process of epicenter regression to *T. cruzi* infection data in Arepuipa, Peru.
(MP4)

Video S4 **Four-Epicenter Animation.** 4 Epicenter Model: Animation describing the Monte Carlo Markov Chain fitting process of epicenter regression to *T. cruzi* infection data in Arepuipa, Peru.
(MP4)
