# Supplementary material for: Retracing Micro-Epidemics of Chagas Disease Using Epicenter Regression
Source: PLoS Comput Biol. 2011 Sep 15;7(9):e1002146. doi: 10.1371/journal.pcbi.1002146 (PMC3174153; doi:10.1371/journal.pcbi.1002146)
Supplement: Alternative Language Text S1 — Spanish translation of the article by Denisse Barbu Covantes. (PDF) [file pcbi.1002146.s001.pdf]

MICROEPIDEMIAS DE LA ENFERMEDAD DE CHAGAS EN AREQUIPA, PERU

Título corto: microepidemias de la enfermedad de Chagas

Palabras clave: Epidemia, urbano, enfermedad de Chagas, Trypanosoma cruzi, Triatoma infestans, Perú, ecología de las enfermedades

Autores:

Michael Z. Levy [1]

Dylan S. Small[2]

Daril A. Vilhena[3]

Natalie M. Bowman[4]

Vivian Kawai[5]

Juan G. Cornejo del Carpio[6]

Eleazar Cordova-Benzaquen[7]

Robert H. Gilman[8]

Caryn Bern[9]

Joshua B. Plotkin[3]

Afiliaciones de los autores

[1] Center for Clinical Epidemiology & Biostatistics, Department of Biostatistics & Epidemiology, University of Pennsylvania School of Medicine, Philadelphia, PA, USA 19104

[2] Department of Statistics, Wharton School, University of Pennsylvania, Philadelphia, PA 19104 USA

[3] Department of Biology, University of Pennsylvania, 219 Carolyn Lynch Laboratories, Philadelphia, PA 19104 USA

[4] Johns Hopkins Hospital, Johns Hopkins University, Baltimore, MD, USA

[5] Asociación Benéfica PRISMA, Lima, Perú

[6] Gerencia Regional del Ministerio de Salud, Av. Daniel Alcides Carrión 505- Cercado Arequipa, Perú

[7] Departamento de Microbiología, de la Facultad de Medicina de la Universidad Nacional de San Agustín, Arequipa, Perú

[8] Bloomberg School of Public Health, Johns Hopkins University, Baltimore, MD, USA

[9]Division of Parasitic Diseases, Centers for Disease Control and Prevention, Atlanta, GA, USA

*Autor corresponsal:

Dr. Michael Levy

819 Blockley Hall
423 Guardian Drive
Philadelfia, PA 19104-6021
Teléfono: 215-898-5300
Fax: 215-573-5315
correo electrónico: mzlevy@mail.med.upenn.edu

Traducido por:

Denisse Barbu Covantes

RESUMEN

La transmisión vectorial de la enfermedad de Chagas se ha convertido en un problema urbano en la ciudad de Arequipa, Perú. No obstante, los síntomas debilitantes que pueden ocurrir en la fase crónica de la enfermedad son rara vez observados en los hospitales de la ciudad. La ausencia de una evidente enfermedad clínica en Arequipa ha llevado a especular que la cepa local del agente etiológico, ha disminuido su patogenicidad crónica.

El largo período asintomático de la enfermedad de Chagas nos lleva a una hipótesis alternativa en ausencia de casos clínicos en Arequipa: la transmisión en la ciudad podría ser tan reciente que la mayoría de las personas infectadas aún no han llegado a la fase tardía de la enfermedad.

En el presente artículo se describe un método nuevo, *epicenter regression*, el cual nos permite inferir la historia espacial y temporal de la transmisión de la enfermedad partiendo de una fotografía instantánea del estado de infección de la población. Se demuestra que en una comunidad de Arequipa, la transmisión de *T. cruzi* ocurrió como series de microepidemias, en las que la más antigua inició hace tan sólo 20 años. Antes de que la transmisión del parásito fuera interrumpida a través de la aplicación de insecticidas en el año 2004, estas microepidemias infectaron a casi el 5% de la comunidad.

En la comunidad estudiada, la mayoría de las infecciones en humanos surgieron en un breve período de tiempo inmediatamente anterior al control de vectores. De acuerdo con nuestros resultados, se espera que los síntomas de la fase crónica de la enfermedad de Chagas no se presenten -aunque la cepa sea patógena en la fase crónica de la enfermedad-, debido al largo período asintomático de la enfermedad y la breve historia de la intensa transmisión.

RESUMEN DEL AUTOR

La enfermedad de Chagas se ha convertido en un problema urbano en la ciudad de Arequipa, Perú, no obstante, hay muy pocas personas que presentan síntomas severos de la enfermedad. Con frecuencia, los síntomas severos no aparecen hasta años después de la infección. Para determinar por qué tan pocas personas presentan síntomas severos, se utilizó un nuevo método, el de epicenter regression, para trazar la historia de la transmisión de la enfermedad de Chagas en una comunidad de Arequipa, Perú.

Nuestros resultados sugieren que en Arequipa, la transmisión ocurrió a través de una serie de pequeñas epidemias; la más antigua de éstas inició hace tan sólo 20 años. Estas microepidemias infectaron a casi el 5% de la comunidad antes de que el insecto portador de la enfermedad de Chagas, *Triatoma infestans* fuera eliminado a través de la aplicación de insecticida. En la comunidad de estudio, la mayoría de las infecciones humanas surgieron durante un breve período de tiempo inmediatamente anterior a la aplicación de insecticidas. Con base a nuestros resultados, se espera que los síntomas severos de la enfermedad de Chagas no se presenten en la comunidad debido a la corta duración de la infección con el parásito, incluso entre los individuos de edad avanzada con la enfermedad de Chagas.

INTRODUCCIÓN

La enfermedad de Chagas, responsable de más muertes en las Américas que cualquier otra enfermedad parasitaria [1], se ha vuelto un problema urbano en la ciudad de Arequipa, Perú. No obstante, los síntomas debilitantes de la enfermedad de Chagas en su fase crónica, comunes en el sur de América del Sur, son rara vez observados en los hospitales de la ciudad (ver información complementaria S1).

La ausencia de una enfermedad manifiesta en los hospitales y en la población de Arequipa en general, ha llevado a los médicos locales a especular que la cepa local del agente etiológico, tiene baja patogenicidad crónica (observación personal), a pesar de que ha causado infecciones mortales agudas en los niños [4] y en animales modelos [5,6].

La gran mayoría de las 8 a 10 millones de personas infectadas por *T. cruzi*, tienen la forma indeterminada de la enfermedad de Chagas. Estas personas no presentan síntomas o signos de la infección, pero del 20% al 30% se espera el progreso a las formas cardíacas o digestivas de la enfermedad de Chagas crónica, que son difíciles de tratar y potencialmente fatales [8-10]. La progresión a la enfermedad cardíaca clínicamente manifiesta es un proceso lento [8,10]. Para la transmisión vectorial, pueden pasar décadas desde el momento de la infección inicial a través del contacto con las heces de un triatomino infectado hasta el inicio de los síntomas. El tratamiento con los medicamentos existentes antitrypanosomal, benznidazol o nifurtimox, parece retardar o prevenir la progresión de la enfermedad [9], pero se piensa que el tratamiento es más eficaz cuando se administra en el curso temprano de la infección [11].

El largo período asintomático de la enfermedad de Chagas nos lleva a una hipótesis alternativa para la ausencia de casos clínicos en Arequipa: la transmisión en la ciudad podría ser tan reciente que la mayoría de las personas infectadas aún no han llegado a la fase tardía de la enfermedad. Para evaluar esta hipótesis es necesario esclarecer el momento de la infección por *T. cruzi* en la población. La fecha de infección por *T. cruzi* es raramente conocida por las personas con la enfermedad de Chagas. La inexistencia de pruebas de factores parasitarios o de huéspedes, cede el paso a pistas fiables en cuanto a la duración de la infección. No obstante, el proceso dinámico de la transmisión de *T. cruzi*, crea patrones espaciales y temporales claros de la infección en los insectos vectores [12-14] y en los huéspedes humanos [15-20].

Tradicionalmente, los análisis de enfermedades infecciosas tienen como finalidad describir los factores de riesgo de infección en un momento estático de la transmisión, utilizando métodos estadísticos para suavizar las heterogeneidades en la exposición entre las personas creadas por la propagación del agente [21-22], o modelar la propagación del agente a través del tiempo y del espacio,

asumiendo una homogeneidad en la población [23]. Trabajos recientes han adoptado un enfoque más unificado, estimando parámetros de propagación y factores de riesgo locales juntos [24-25]. Haciendo algunas excepciones notables, especialmente [26-27], el análisis más unificado de la propagación de la enfermedad y los factores locales de riesgo se dirige a situaciones en las que se conoce la hora y el lugar de la introducción del agente patógeno. Aquí se desarrolla un modelo de regresión, el de *epicenter regression*, con el objetivo de inferir la propagación temporal y espacial de un agente patógeno en la situación más común en la cual se desconoce el sitio o los sitios de la introducción de un agente de la enfermedad.

Trypanosoma cruzi es un parásito de movimiento lento. Cuando se transmite en una fase epidémica, el período de tiempo durante el cual se expone cada individuo a la infección varía en gran medida según lo lejos que él vive del sitio de introducción del parásito. La heterogeneidad en cuanto al tiempo de exposición que resulta de la propagación lenta del parásito puede resultar en una agrupación espacial observable de las infecciones. Empero, la agrupación espacial observada, también puede surgir en la transmisión endémica si los factores locales de riesgo de infección se concentran en ciertas áreas.

Los modelos de *epicenter regression* son explícitos en cuanto a la duración de la exposición de un individuo en función de la distancia de su hogar a un sitio (desconocido) de introducción de la enfermedad, por lo que, dado el tiempo de exposición de los hogares de cada individuo, estiman el efecto de los factores de riesgo medidos en el hogar respecto a la probabilidad de infección para cada individuo. Los modelos se ajustan a los patrones de infección por *T. cruzi* en insectos vectores y en huéspedes humanos en la comunidad peri-urbana de Guadalupe, Arequipa, Perú [3,15].

Se utilizaron métodos Bayesianos y el método Monte Carlo con cadenas de Markov (MCMCs), enfoques que nos permiten hacer inferencias sobre los parámetros que reflejan la incertidumbre de factores desconocidos, tales como la localización de casos en un proceso infeccioso dinámico [26-28].

Se consideraron modelos con un solo sitio de introducción de *T. cruzi*, así como los modelos con múltiples sitios y momentos de introducción llevando a múltiples microepidemias. Por microepidemia se refiere a un foco de transmisión, sembrado desde la introducción misma del parásito, la cual es discreta y discernible de la gran tendencia de transmisión de la comunidad. También se comparan estos modelos al modelo endémico, en el cual se asume que cada individuo es expuesto al parásito desde el nacimiento. En el modelo endémico la agrupación de infección se explica principalmente por la agrupación de factores de riesgo de los hogares.

Utilizando las estimaciones de estos modelos, se calcula la prevalencia de infección estimada entre la población para cada año hasta la interrupción de la transmisión a través de la aplicación de insecticida en el año 2004. Se discute cómo un conocimiento más preciso de la historia de transmisión

podría explicar la ausencia de la fase tardía de la enfermedad de Chagas en Arequipa, y potencialmente informar acerca del manejo clínico de los individuos con la enfermedad de Chagas indeterminada.

MÉTODOS

Encuesta transversal en la comunidad

Se llevaron a cabo encuestas transversales entomológicas y serológicas en un asentamiento reciente (Pueblo Joven), Guadalupe, en el margen suroccidental de la ciudad de Arequipa, Perú [14]. La transmisión vectorial de la enfermedad de Chagas en Guadalupe fue interrumpida por la aplicación de insecticida, deltametrina, en noviembre de 2004. Paralelamente a la aplicación de insecticidas, la presencia y la densidad del vector se determinaron a través de una búsqueda hora/hombre por profesionales capacitados. Triatominos adultos y de quinto estadio, vivos y moribundos fueron examinados consecutivamente en cada sitio para determinar la presencia de *T. cruzi* hasta encontrar 1 insecto con resultado positivo; 10 insectos con resultado negativo o todos los insectos disponibles, lo que ocurriera primero [14]. En Agosto de 2005, todos los residentes de la comunidad fueron invitados a participar en una encuesta serológica para detectar la enfermedad de Chagas. Las localizaciones de todos los hogares en Guadalupe se determinaron con un sistema manual de posicionamiento global (Garmin Corporation, Olathe, KS, EE.UU.A). Los sueros fueron examinados utilizando un kit comercial de ensayo de inmunoabsorbencia ligado a enzimas (ELISA) con un antígeno de lisado de epimastigotes (Chagatek, Biomerieux, Buenos Aires, Argentina) siguiendo las instrucciones del fabricante. Los ELISAs positivos fueron confirmados por el ensayo de anticuerpos de inmunofluorescencia indirecta (IFI) siguiendo los métodos estándar; un título de 1:32 se consideró positivo. El análisis estadístico de los datos provenientes de Guadalupe fue aprobado por el Comité de Revisión Institucional de la Universidad de Pensilvania.

Modelo de epicenter regression

Epicenter regression infiere en dónde y cuándo el agente de la enfermedad fue introducido en una comunidad y como covariables medidas dentro del hogar afectan el riesgo de infección dada la exposición. Se inicio con un modelo simple [29], en cual un individuo tiene un riesgo constante de ser infectado una vez expuesto. La probabilidad de que un individuo este infectado es igual a 1 menos la probabilidad de que el individuo escape de la infección:

$$1 - e^{-\text{Riesgo una vez expuesto} \, * \, \text{Duración de la exposición}}$$

Esta ecuación se conoce como el modelo catalítico, ya que también describe la probabilidad de un cambio en el estado de las moléculas expuestas a un constante bombardeo de un catalizador [30-31]. Se amplia este marco en un modelo biológico plausible al permitir al "Riesgo una vez expuesto" y a "la duración de la exposición" variar entre los individuos en función de las covariables observadas y estimar el efecto de las covariables en los datos observados. Se estimo el riesgo de las personas expuestas, ***i***, debido a las covariables medidas en sus hogares, ***j***, utilizando un método tradicional para estimar el riesgo, un modelo log-lineal: $Riesgo_i = e^{\alpha + \sum \beta X_i}$. Donde, X_i representa un vector de covariables medido en cada individuo, y el parámetro β describe cómo estas covariables aumentan o disminuyen el registro del riesgo para cada individuo viviendo en el hogar expuesto. Se asume que el efecto de cada covariable es constante, y no varía año con año ni localización a localización. El punto de intersección, ***α***, denota la base del riesgo de los individuos expuestos cuando todas las covariables tienen un valor de cero.

Se examinaron las covariables antes presentadas para ser asociadas con la infección por *T. cruzi* en niños de la comunidad, incluyendo: la presencia de animales, casi exclusivamente de perros y gatos, durmiendo en el ámbito doméstico de la familia y el número de vectores al interior del hogar durante la búsqueda entomológica programada [15].

Para cada individuo se estimó el período de tiempo durante el cual él o ella habían estado expuestos a *T. cruzi* por el menor de dos correlatos de exposición fácilmente observados o estimados: la edad del individuo y la duración de la exposición de su hogar. Se asume que el tiempo de exposición de un hogar es una función de tres parámetros observados: ***T***, es el tiempo transcurrido desde la introducción de *T. cruzi* en la comunidad, ***d***, la distancia del hogar en el que se introdujo el primer parásito, y, ***r***, la velocidad de propagación del parásito.

En el presente análisis, se supone que un hogar se expone durante un tiempo proporcional a su distancia del sitio de introducción del parásito. Este supuesto es una simplificación común de análisis

matemático de los organismos invasores basado en ecuaciones de difusión, las cuales concluyen que, cuando se asume que los individuos se mueven de acuerdo a recorridos aleatorios, la tendencia principal de la población avanza a un ritmo constante del sitio de introducción [23].

El tiempo después de la introducción hasta que el parásito alcanza a la familia, durante el cual los residentes del hogar no están expuestos es: d_{jk}/r , y la duración total de la exposición es $T- d_{jk}/r$, donde d_{jk} es la distancia del hogar j desde el lugar de introducción k. La probabilidad de infección del individuo i en el modelo es :

$$1-e^{-(e^{\alpha+\sum\beta X_i})\bullet(T-d_{jk}/r)}$$

para todos los individuos nacidos antes de que su hogar estuviera expuesto a los vectores portadores del parásito, y:

$$1-e^{-(e^{\alpha+\sum\beta X_i})\bullet edad_i}$$

para todos los individuos nacidos después de que su hogar fuera expuesto a los vectores portadores del parásito. En el área de estudio la cima de una colina separó los hogares. Se calculó la distancia entre los hogares alrededor de esta cima. Como en el relacionado modelo de riesgos proporcionales [32], el tiempo de exposición en *epicenter regression* debe limitarse a valores positivos. En el modelo anterior, si d/r, es mayor que T, entonces el tiempo de exposición se ajusta a cero y la probabilidad de infección es igual a cero. Se utilizaron datos sobre la presencia de *T. cruzi* en insectos para refinar nuestras estimaciones de los parámetros T, d, y r. Para los regímenes de los parámetros en los que el modelo no predijo ninguna exposición a los hogares donde se había observado *T. cruzi* en vectores, se determinó una *likelihood* de cero.

Introducciones Múltiples

Se expandió nuestro marco de *epicenter regression* para considerar la posibilidad de que *T. cruzi* se introdujo en Guadalupe en múltiples ocasiones. Cada introducción se produciría en un hogar diferente, k, y en un momento diferente, T_k. Se calculó la duración de la exposición de los individuos en el hogar j debido a la introducción en los hogares k como: $T_k - (d_{jk}/r)$. Se asumió que una vez que la transmisión se estableció en un hogar permanece asentada. También se supone que la introducción de parásitos adicionales en un hogar en el que la transmisión ya se ha establecido, no incrementa el riesgo de infección entre las personas que viven en el hogar. La duración de la exposición de cada individuo, es por lo tanto el máximo de su exposición para cada introducción, o bien, su edad si su hogar fue

expuesto antes de su nacimiento. La probabilidad de infección de cada individuo es de otra manera según lo calculado anteriormente.

Modelo endémico

Con el propósito de comparar, se adaptó un modelo endémico, en el cual se asume que el tiempo de exposición de cada individuo fue equivalente a su edad. La probabilidad de infección del individuo *i* viviendo en el hogar *j* bajo el modelo endémico es:

$$1 - e^{-(e^{\alpha + \sum \beta x_i}) \bullet edad_i}$$

Incorporación de conocimientos previos

El análisis Bayesiano de *epicenter regression* comienza con lo que se conoce de la epidemia antes de realizar la prueba en las personas de la comunidad, conocidos como los "*priors*" del modelo [33]. Un supuesto clave del modelo es que el parásito se introduce en un hogar y se extiende desde el sitio de introducción. Antes de observar los datos que no tienen información acerca de en cual hogar (u hogares) el parásito se introdujo por primera vez y así establecer una distribución previa uniforme de la probabilidad de que cada hogar era un sitio de introducción. Se establecieron *priors* no informativos (Gaussian, con una media de cero y una desviación estándar de 10^3) sobre el efecto de las covariables a nivel de los hogares (los βs).

La comunidad de Guadalupe era de 40 años de edad en el momento de la recolección de datos. Por lo tanto, se estableció un *prior* uniforme entre 1 y 40 años del tiempo transcurrido desde la introducción del parásito, para cada introducción, T_k.

Se proporcionó un *prior explícito* en la velocidad de propagación del agente patógeno permitiendo así la adaptación del análisis a las particularidades de *T. cruzi* transmitido por *T. infestans.* El *prior* se basó en la velocidad de propagación del frente de onda de *T. cruzi* de datos longitudinales de Villa la Joya, una comunidad similar a la de Guadalupe en términos de densidad de los hogares, de su historia y de las prácticas en cría de animales. En Villa la Joya encuestamos a 30% de los hogares en enero de 2008, descubriendo seis sitios de infección de *T. cruzi* entre los insectos triatominos. Se realizó una encuesta exhaustiva aunada a la aplicación de insecticida por el ministerio de salud, siguiendo la metodología utilizada en Guadalupe, en noviembre y diciembre de 2008.Durante la

encuesta exhaustiva, se identificaron cuarenta y cuatro focos adicionales de *T. cruzi* en vectores, grupos bien definidos alrededor de los sitios pre-existentes donde se encontraba la presencia de parásitos. Asumiendo que el parásito se había extendido desde el centro de cada grupo, habría viajado aproximadamente 18 metros a lo largo del intervalo de 10 a 12 meses, entre los estudios preliminares y las encuestas exhaustivas, y finalmente llegado a cada uno de los 44 focos. En ningún caso el parásito se habría extendido más allá de 58 metros de un sitio ya existente.

Basándose en estos hallazgos, se asignó una distribución normal, con una media de 20 metros y una varianza de 100 metros, para describir la probabilidad *prior* de la velocidad anual de propagación del parásito en Guadalupe. Una tasa de propagación de 20 metros/año es semejante a un solo hogar infestado que infesta del parásito en promedio entre 6 y 7 hogares adicionales al año cuando se introduce en una comunidad susceptible.

Fitting, comparación y predicción del Modelo

Se adaptaron los modelos de *epicenter regression* utilizando los métodos Bayesianos y el Método Monte Carlo con cadenas de Markov (MCMCs, por sus siglas en inglés). Se actualizaron los MCMCs utilizando los algoritmos Metrópolis y de Metrópolis-Hastings [33] (véase el comentario del código en el apéndice técnico: Texto S3, S1 conjunto de datos, Texto S4, Texto S5). Para el modelo endémico y modelos con 1 a 10 introducciones, se realizaron 50 replicas de las cadenas de MCMC, cada una con 1 millón de estimaciones. Se descartó el primer 100.000, reteniendo cada 10ª estimación en el resto de la cadena, para reducir la autocorrelación entre las estimaciones.

Para cada par de modelos comparados, se estimó el factor de Bayes con el promedio, en los 50 pares de cadenas, del ratio de medias armónicas de la probabilidad posterior para los modelos [34]. Se aseguró la convergencia de las cadenas con la prueba de Geweke [35] y con el estadístico Gelman-Rubin [36]. Se consideraron los modelos con 1 a 10 introducciones parasitarias en la población (Ver películas en materiales complementarios: Texto S2, Video S1, Video S2, Vídeo S3, Video S4). Se estimó la prevalencia de cada año en modelos alternativos, integrando el riesgo de infección por unidad de tiempo, durante el período de tiempo que cada individuo fue expuesto.

Por último, se cuantificó el número esperado de casos de la enfermedad de Chagas en etapa tardía entre los individuos infectados con *T. cruzi* en el momento del estudio. El tiempo entre la infección y el desarrollo de la enfermedad en etapa tardía no es certero, pero en general se estima entre 10 y 30 años [8]. Se utilizan tres modelos alternativos para describir la probabilidad de aparición de la enfermedad en etapa tardía como una función de tiempo:

1.Una distribución de Poisson centrada en 20 años;
2.Una distribución de Gauss con una media de 20 años y 95% de densidad probable entre 10 y 30 años;
3.Una distribución uniforme limitada a 10 y 30 años.

Para cada uno de estos se integró la dinámica temporal predicha y se calculó el número esperado de casos, y la probabilidad de observar cero casos con exactitud. Se utilizó una estimación altamente conservadora de la proporción de individuos que eventualmente desarrollarán la enfermedad en etapa tardía, el 30% [8].

RESULTADOS

Los modelos que describen la transmisión de *T. cruzi* como epidemia se adaptan a los datos recolectados en la comunidad de Guadalupe mucho mejor que el modelo endémico. Las probabilidades a favor de los modelos epidemiológicos en comparación con el modelo endémico aumentaron más de 4 órdenes de magnitud después de considerar los datos observados, es decir, los factores de Bayes que comparan el modelo epidemiológico con el endémico superaron 10^4 (Figura 1). Un factor de Bayes mayor que 10 es considerado una "evidencia sólida" a favor de un modelo sobre otro [34].

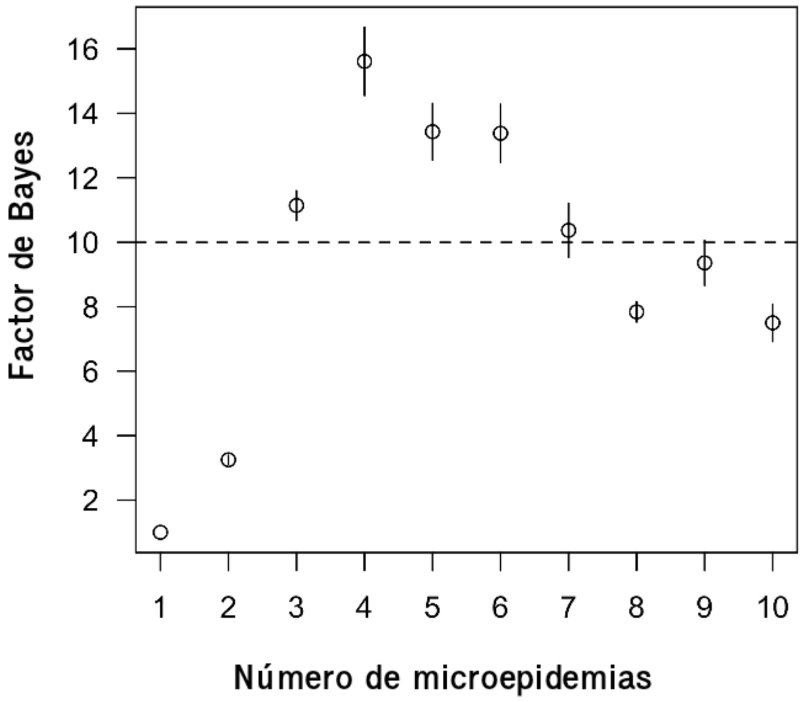

Figura 1. Comparación del ajuste de los modelos multi-epidémicos para describir la transmisión de *T. cruzi* en Guadalupe, Arequipa, Perú, para un modelo epidémico. Se muestran el error medio y el error estándar de los factores de Bayes estimados "para la comparación de cada modelo con el modelo de un epicentro". La línea punteada indica los modelos con un fuerte sustento en relación con el modelo epicentro 1 (factor de Bayes >10).

Los modelos que describen la transmisión de *T. cruzi* como series de microepidemias focalizadas fueron mejor fundamentados que un modelo con una sola epidemia derivada de un sitio de introducción. En particular, el modelo con cuatro microepidemias tuvo mayor sustento de los datos observados; las probabilidades a favor de este modelo en comparación con el modelo de una sola epidemia aumentó de 15 veces después de considerar los datos observados (Figura 1).

De acuerdo con el modelo de cuatro epicentros, el parásito se introdujo por primera vez en Guadalupe hace 20 años. Cuando se tabuló el tiempo de exposición y el riesgo de infección de individuos en la población en el modelo de cuatro epicentros, se encontró que alrededor de la mitad de las infecciones ocurrieron en los 5 años anteriores a la interrupción de la transmisión, a través de la aplicación de insecticidas, y el 90% de las infecciones ocurrieron en un período de 12 años (Figura 2). Estas estimaciones fueron consistentes entre los modelos con 2 a 10 epicentros (Figura 2). En contraste, bajo el modelo endémico, la prevalencia aumentó lentamente en un período de tiempo mucho más largo.

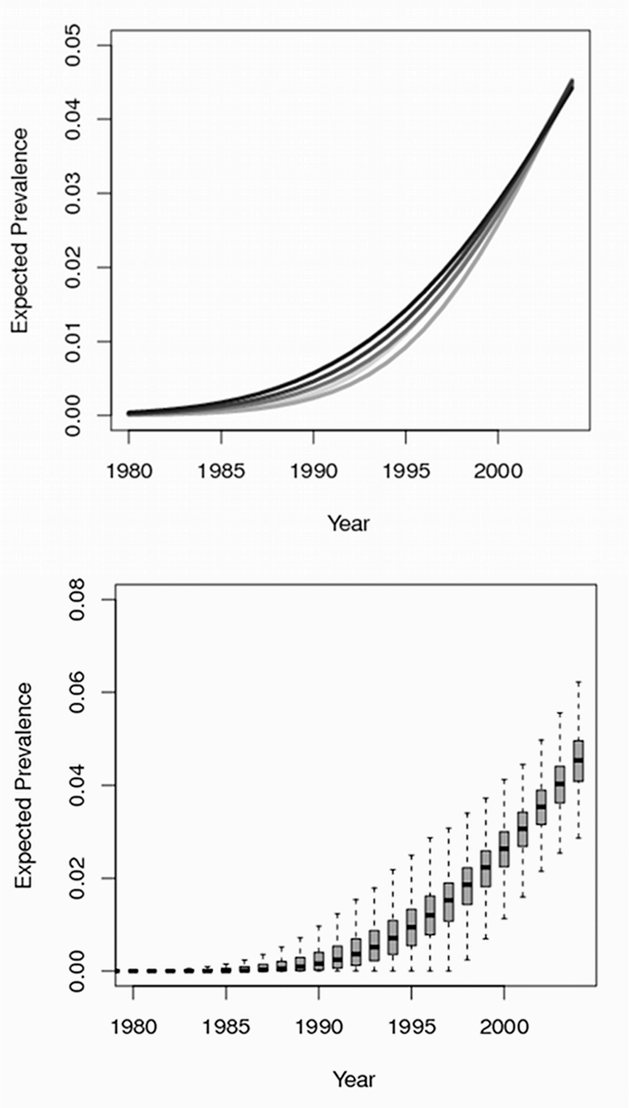

Figura 2. El porcentaje esperado de participantes del estudio infectados por cada año posterior a 1980.
A. La expectativa para los modelos con 2,4,6,8 y 10 epicentros, las líneas están sombreadas en función del número de epicentros (2 epicentros=gris claro, 10 epicentros=negro).

B. Un diagrama de caja que muestra los intervalos creíbles y de la mediana para las estimaciones posteriores para el mejor ajuste del modelo de regresión para cuatro epicentros.
La enfermedad de Chagas es una infección de por vida, los individuos infectados se asumen permanecer seropositivos a lo largo de sus vidas.

Espacialmente, la primera introducción en el modelo de los cuatro epicentros se produjo en el suroeste de la comunidad (Figura 3). Una segunda microepidemia brotó en el noroeste de la comunidad, seguido de una tercera en el sureste. Las estimaciones sobre la posición de la cuarta microepidemia variaron, algunas se centraron en un hogar con vectores transmisores de *T. cruzi* en el extremo este de Guadalupe, mientras que otros

estaban más cerca a un hogar con un caso humano en el extremo norte de la comunidad. Los modelos con más de cuatro introducciones generalmente subdividieron estas microepidemias, tales divisiones disminuyeron el sustento estadístico para estos modelos.

Figura 3. La posición geográfica estimada de las introducciones de *Trypanosoma cruzi* en la comunidad de Guadalupe, Arequipa, Perú.

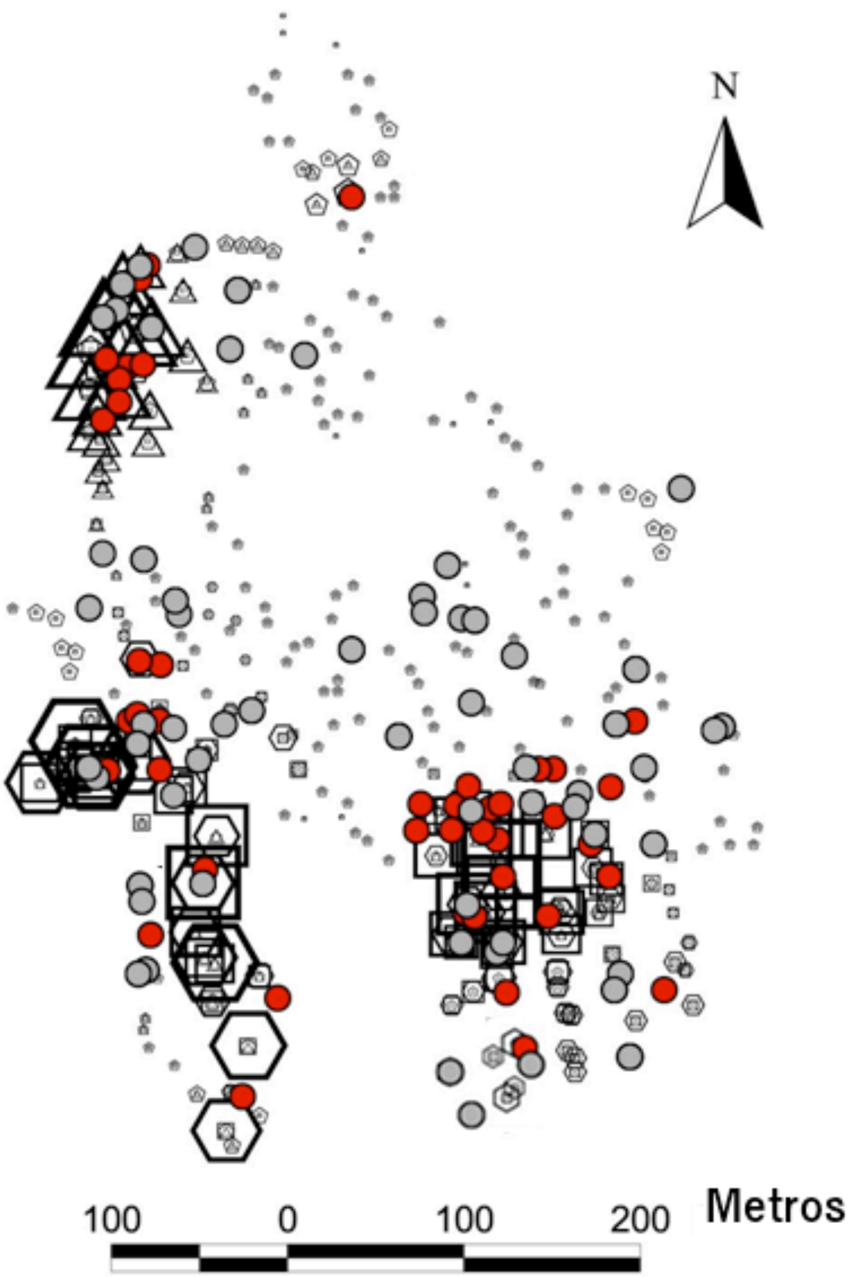

La probabilidad de que cada hogar fue el primero (hexágonos), segundo (triángulos), tercero (cuadrados) o cuarto (pentágonos) lugar de introducción en el modelo de los cuatro epicentros es representada. Formas más grandes corresponden a mayores probabilidades. Los hogares con casos de la enfermedad en humanos se representan con círculos rojos, y aquellos de vectores portadores de *T. cruzi* se representan con círculos de color gris.

Los pasos siguientes se tomaron para proteger el anonimato del paciente en la toma de este mapa: la posición geográfica de los casos humanos ha sido modificada ligeramente y al azar; las posiciones de algunos hogares no infectados han sido alteradas, y algunas regiones del mapa se han girado en ángulo al azar alrededor de su centro de gravedad.

Temporalmente, el modelo de cuatro epicentros capturó la relación entre la edad y la prevalencia observada en los datos (Figura 4). Las estimaciones del momento de la primera introducción de *T. cruzi* en la comunidad fueron sorprendentemente similares en los modelos epidemiológicos (hace 19.98 años en el modelo de un epicentro, 20.31 en el modelo de cuatro epicentros). El efecto estimado de las covariables sobre el riesgo de infección dada la exposición

también fue similar, con un incremento del riesgo de 1-2% por insecto capturado en la zona doméstica de un hogar y un aumento de riesgo de 40-60% entre los individuos expuestos que permitieron a los animales dormir de noche en el interior del ámbito doméstico del hogar (Tabla 2).

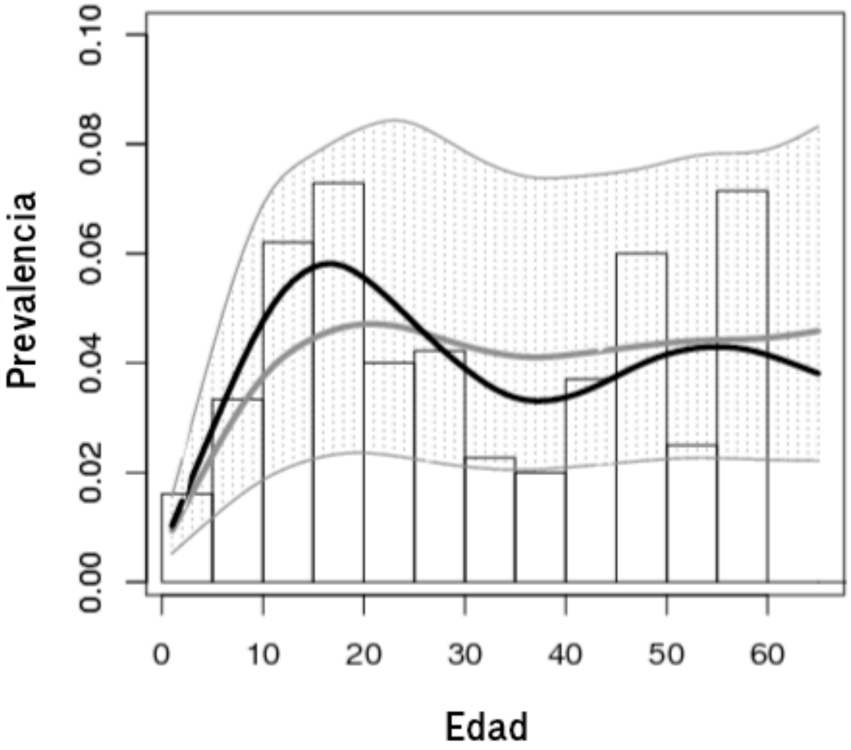

Figura 4. La relación observada y esperada entre la edad y la prevalencia de infección de *Trypanosoma cruzi* en Guadalupe, Arequipa, Perú. El histograma representa los datos observados; una ranura suavizada, ponderada por el número de observaciones para cada edad, está ajustada a estos datos (curva de color negro). Las estimaciones del modelo en cuanto a la relación entre la edad y la prevalencia se calcularon mediante la determinación de la probabilidad de infección para cada individuo derivada de las predicciones posteriores del modelo *epicenter regression* con cuatro epicentros. El ajuste de la línea a las predicciones posteriores de la mediana se encuentra rodeado por una región limitada por las líneas ajustadas a las predicciones del 2,5% y 97,5% de los cuantiles de la parte posterior (de color gris claro, sombreado).

Cuadro 1. Estimaciones posteriores e intervalos de credibilidad para endemia, epidemia única y modelo de cuatro microepidemias de trasmisión de *Trypanosoma cruzi* en la zona periurbana de Arequipa.

Parámetro	Modelo endémico	Modelo epidémico único	Modelo de cuatro microepidemias
	Mediana [2.5%, 97.5% cri[1]]	Mediana [2.5%, 97.5% cri[1]]	Mediana[2.5%, 97.5% cri[1]]
Tasa de propagación del parásito (mes / año)		29.18 [17.52 - 44.69][2]	17.35 [8.87 - 32.55][3]
Línea de base anual de riesgo de infección	0.0014 [0.0009 - 0.0020]	0.0032 [0.0018 - 0.0058]	0.0042 [0.0022 - 0.0086]
Riesgo relativo por insecto domiciliario capturado	1.018 [1.004 - 1.028]	1.014 [1.000 - 1.024]	1.014[1.000 -1.025]
Riesgo relativo en los hogares con animales durmiendo en el interior	1.34 [0.66 - 2.56]	1.53 [0.76 - 2.93]	1.42 [0.70 - 2.72]
Años desde la primera introducción del parásito		20.31 [12.71 - 33.25]	19.98 [10.92 – 34.65]

[1] Intervalos de credibilidad son los 2.5% y 97,5% cuantiles de las muestras posteriores.

[2] Corresponde a un promedio de 15.01 [5.07, 34.26] hogares expuestos por un sólo hogar infectado en una población totalmente susceptible.

[3] Corresponde a un promedio de 4.950 [1.01, 18.77] hogares expuestos por un sólo hogar infectado en una población totalmente susceptible.

Sin embargo, las estimaciones posteriores sobre el índice de propagación de *T. cruzi* variaron en gran medida. En el mejor ajuste del modelo de cuatro epicentros un sólo hogar expuesto expondría en promedio a 4-5 hogares adicionales por año en una comunidad propensa. Por el contrario, en el modelo de un epicentro, la tasa estimada de propagación expondría a un número exageradamente grande de hogares (en promedio 15 hogares por año en una comunidad propensa). La tasa de propagación estimada más alta fue contrarrestada por un riesgo menor estimado de infección dada la exposición en el modelo de un epicentro en comparación con el modelo de cuatro epicentros.

Cuando se combinaron nuestras estimaciones posteriores del modelo, respecto a la fecha de la infección por *T. cruzi*, con tres modelos alternativos de la distribución de los tiempos de espera, entre la

infección parasitaria y el desarrollo de la etapa tardía de la enfermedad de Chagas, se encontraron probabilidades muy elevadas de observar menos de un caso de enfermedad en etapa tardía en Guadalupe en el momento de la encuesta (Figura 5). En el marco del modelo de cuatro epicentros las probabilidades de observar menos de un caso de enfermedad en etapa tardía fueron 0.696, 0.937, 0.967 bajo el modelo de Poisson, de Gaussian y el modelo uniforme, respectivamente; dadas las estimaciones posteriores de la fecha de infección *T. cruzi*, de acuerdo con el modelo de un epicentro, las probabilidades de observar menos de un caso fueron 0.797, 0.763, 0.779 en los modelos de Poisson, de Gaussian y el uniforme, respectivamente.

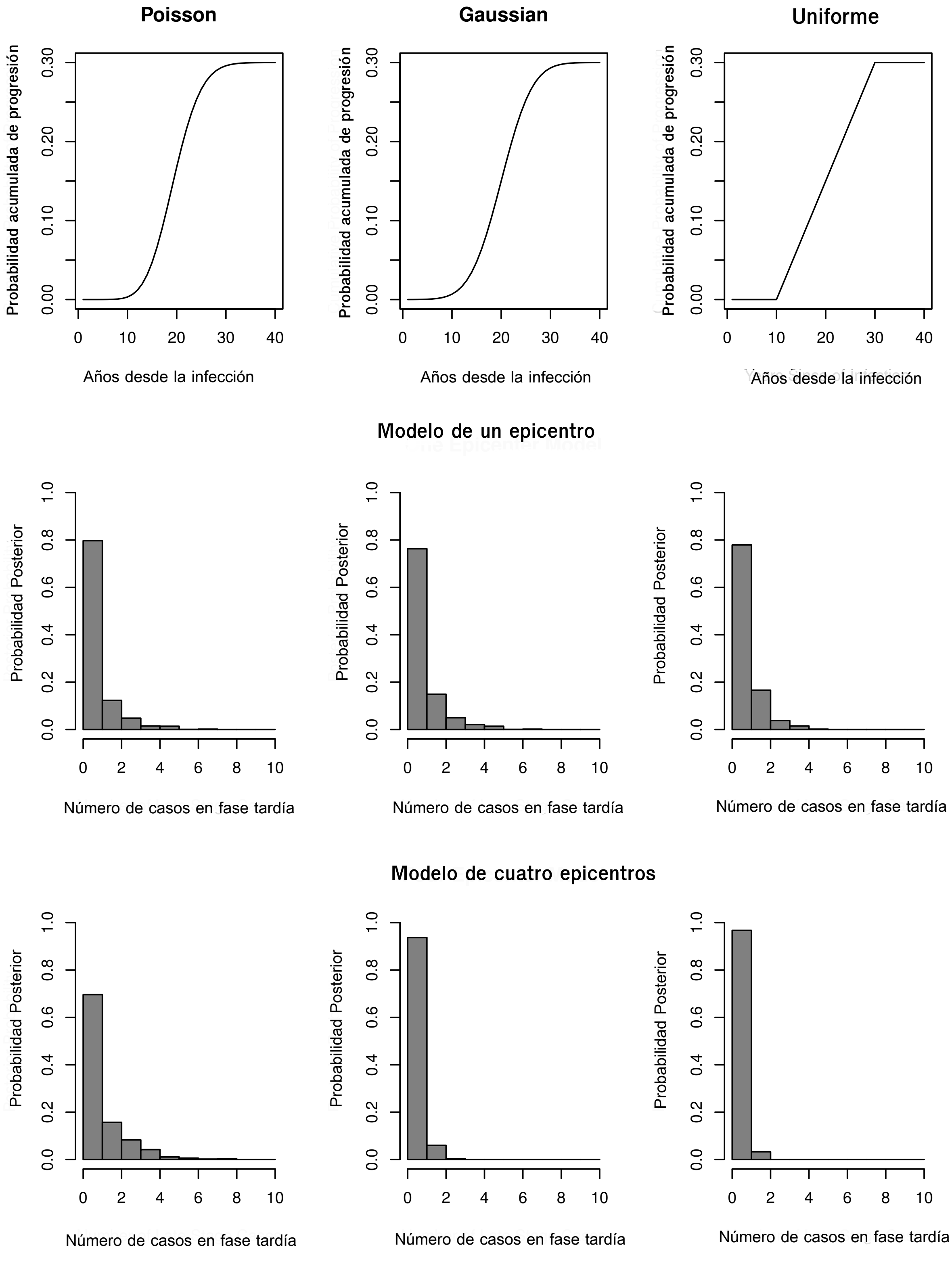

Figura 5. El número esperado de casos de la enfermedad de Chagas en etapa tardía entre los individuos infectados con *T. cruzi* en Guadalupe, Arequipa, Perú en 2004. Fila superior: tres modelos alternativos para describir la probabilidad de aparición de la enfermedad en fase tardía en función del tiempo (ver texto). Los histogramas representan el número posterior deseado de casos en fase tardía en el modelo de cuatro epicentros (Fila central) y el modelo de un epicentro (fila inferior).

DISCUSIÓN

La transmisión de *Trypanosoma cruzi* en la zona periurbana de Arequipa ocurre en series de microepidemias espacialmente focalizadas. En la comunidad de Guadalupe, la más antigua de estas microepidemias surgió hace tan sólo unos 20 años. Para el tiempo en el que la transmisión vectorial de *T. cruzi* fue interrumpida a través de la aplicación de insecticida en el año 2004, la prevalencia de infección humana había alcanzado el 5% y fue incrementando rápidamente. La prevalencia de infección relativamente elevada parece estar en conflicto con la escasez de pacientes con miocardiopatía chagásica en los hospitales locales, llevando a algunos a concluir que la cepa del parásito en la región tiene patogenicidad crónica baja. Sin embargo, hemos demostrado que la mayoría de las infecciones en Guadalupe probablemente ocurrieron durante un breve período de tiempo previo a la aplicación de insecticida. Nuestros resultados sustentan una explicación diferente de la ausencia de etapa tardía de la enfermedad de Chagas en la ciudad: los daños causados por el vector y el parásito pueden ausentarse porque la mayoría de las personas aún tienen que pasar del largo período asintomático de la enfermedad de Chagas al periodo sintomático.

Nuestros resultados no refutan la hipótesis de que los parásitos que circulan en Arequipa son menos patógenos que otras cepas. Estudios previos sugieren que la cepa de *T. cruzi* en Arequipa es de diversidad genética limitada, posiblemente debido a un efecto fundador [37-38], un hallazgo, que de ser cierto, sería coherente con los resultados presentados aquí. Se han tipificado quince aislados de *T. cruzi* de Arequipa siguiendo los métodos descritos [39]. La mayoría de las cepas, aunque no todas, son *T. cruzi* tipo I, incluyendo los aislados de las comunidades vecinas de Guadalupe [Vitaliano Cama, comunicación personal]. Es absolutamente posible que las cepas particulares de *T. cruzi I* que predominan en Arequipa causen menos patología crónica. Sin embargo, enfatizamos que, basándonos en los resultados de nuestros modelos, la falta de miocardiopatía asociada con la enfermedad de Chagas en la zona periurbana de Arequipa no puede considerarse como evidencia de un parásito débil, y que, en ausencia de tales pruebas, se debe estar preparado para una creciente carga de trabajo en los próximos años relacionada con la enfermedad clínica en la región.

Clínicamente, nuestros resultados pueden contribuir a la reevaluación de las directrices de tratamiento para la enfermedad de Chagas indeterminada que se está llevando a cabo [40-41]. Generalmente, se piensa que el tratamiento farmacológico es más efectivo al inicio del curso de infección por *T. cruzi* [11]. Actualmente, muchos países, incluido Perú, no suelen ofrecer tratamiento a los pacientes mayores de 15 años de edad [1]. Una justificación de esta política se basa en el supuesto de que la edad es un buen sustituto de la exposición al parásito, y un sustituto razonable de la duración de la infección entre los casos. Es decir, en ausencia de pruebas en contra, se supone que las personas

mayores han estado llevando el parásito durante muchos años, y por lo tanto a ser menos probable que beneficien del tratamiento farmacológico. Esta suposición pudiera ser cierta cuando la transmisión sigue un patrón endémico. Sin embargo, cuando la transmisión ocurre en una epidemia, o múltiples microepidemias, la suposición es incorrecta. En la zona periurbana de Arequipa y otras situaciones de epidemia, la edad por sí sola no es un sustituto válido de la exposición. La información temporal y espacial en su conjunto dan una mejor idea de cuánto tiempo un individuo ha sido expuesto a *T. cruzi.*

Geográficamente, nuestro hallazgo de que *T. cruzi* se introdujo por primera vez en el suroeste de Guadalupe tiene un sentido intuitivo. Guadalupe es una península, rodeada por campos a sus tres lados, los cuales no proporcionan buen hábitat para triatominos, mientras que el suroeste de la frontera de la comunidad esta rodeada de una colina grande con asentamientos similares con transmisión de la enfermedad de Chagas documentada [3]. Una vez que *T. cruzi* se introdujo en el suroeste de Guadalupe es claro que no se extendió de hogar en hogar a través de un proceso de difusión simple. En cambio, el parásito se transportó a otros sitios en la comunidad, y ahí sembró nuevas microepidemias de transmisión. Sólo podemos adivinar el mecanismo de transporte del parásito. Los animales, especialmente los cobayos de india (cuyes), son comúnmente un obsequio o comercializados tanto dentro como entre las comunidades, los cuyes infectados podrían haber provocado nuevas microepidemias en Guadalupe. Insectos infectados también pueden volar y establecer nuevos focos de transmisión, mientras que la probabilidad de que cualquier insecto establezca una transmisión es muy pequeña [42], teniendo en cuenta el gran número de insectos en dispersión, el asentamiento podría ocurrir ocasionalmente. En las historias de migración humana, los datos a nivel individual podrían permitirnos estudiar si alguna persona infectada fue quién probablemente trajo el parásito a Guadalupe desde otro lugar [43]. Nuestros resultados acerca de una historia muy reciente de las infecciones fueron robustos en cuanto a la exactitud del número de microepidemias, y es poco probable que sean afectados si estas microepidemias se iniciaron a través de un mecanismo en lugar de otro.

En general, nuestro enfoque es aplicable a cualquier situación en la que la observación esperada de un organismo en los lugares de muestreo, en un momento determinado, es una función del sitio o de los sitios (desconocido/s) de la introducción del organismo en el sistema. La relación funcional entre la expectativa en un sitio de muestreo al sitio(s) de introducción, puede ser una simple función de la distancia, como se ha utilizado aquí, o puede incluir información sobre el hábitat entre los sitios de muestreo y los de introducción. El método puede ser informativo cuando está disponible alguna información previa sobre el avance del agente de la enfermedad o los sitios probables de la introducción. En ausencia de ambos no es probable que el método sea informativo.

Nuestra aplicación de *epicenter regression* para la transmisión de *T. cruzi* simplifica lo que es,

en realidad, un ciclo de transmisión complejo, y es limitado por la naturaleza transversal de los datos. Nuestra información previa sobre la velocidad de propagación del parásito provino de observaciones empíricas de otra parte de Arequipa; sin embargo la naturaleza inherentemente estocástica de dispersión biológica cuestiona nuestra capacidad para extrapolar nuestras observaciones de una comunidad a otra [44]. La medición del factor de Bayes también está limitada por los errores de estimación [34]. Nuestro objetivo principal fue de estimar el momento de infección de *T. cruzi* a diferencia de estimar el número exacto de introducciones del parásito en la comunidad.

Aunque el modelo de cuatro microepidemias fue firmemente sustentado sobre el modelo de un solo epicentro, y de forma sustancial respaldado en el modelo de dos epicentros, hubo poca diferencia en el ajuste entre los modelos con 3-10 microepidemias. Otros métodos, tales como el de MCMC de salto reversible [45], podría ser más eficiente cuando el número de introducciones es de interés primario [28].

El presente estudio se concentró en una sola comunidad peri-urbana. Desde la conclusión de este estudio hemos observado patrones similares de microepidemias de transmisión de *T. cruzi* en los datos entomológicos a través de la mitad sur de la ciudad de Arequipa (datos no publicados). En más zonas rurales fuera de la ciudad, la transmisión de *T. cruzi* fue interrumpida a mediados de los años noventas [46]. Existen otros informes anecdóticos adicionales acerca de la ausencia de la fase tardía de la enfermedad entre los individuos con la enfermedad de Chagas que residen fuera de la ciudad. La ausencia de la etapa tardía de la enfermedad entre los individuos infectados hace muchas décadas, podría proporcionar evidencia de baja patogenicidad crónica. Por el contrario, un resultado de mayor prevalencia de la enfermedad en etapa tardía entre tales individuos podría aportar evidencia directa contra esta hipótesis.

Los patrones tradicionales endémicos de transmisión de *Trypanosoma cruzi* por *Triatoma infestans* se han alterado en gran medida en el sur de América del Sur a través de un programa concertado para el control de vectores conocido como la Iniciativa del Cono Sur [1]. Actualmente, la iniciativa es retada por vectores y parásitos que regresan a las zonas anteriormente controladas con insecticidas [13,40-42], y por nuevos focos de transmisión en y alrededor de los centros urbanos [14,15].

Las microepidemias de transmisión de la enfermedad de Chagas que observamos en Arequipa puede ser un proceso típico de aparición o reaparición del vector y el parásito, en lugar de un patrón anómalo. Distinguir entre la epidemia y la transmisión endémica permitirá una mejor comprensión de la relación dinámica entre la prevalencia de infección por *T. cruzi* y la carga clínica de la enfermedad de Chagas.

AGRADECIMIENTOS

Agradecemos a Michelle Kaplinski y Alicia Hedron quienes supervisaron los estudios basados en el hospital que se debaten en el material complementario; a Vitaliana Cama y a Jenny Ancca Juárez quienes tipificaron las cepas de *T. cruzi* de Arequipa; a F. Ellis McKenzie, Mark Miller, Manuela Verástegui, Viviana Pinedo Cancino, Seitz Amy, Víctor Machaca Quispe, César Náquira, Málaga, Fernando, Frank Steurer, Christenson Megan, Kate Levy, Corentin Barbu y a todos los que ayudaron en el territorio de Arequipa y en el laboratorio de Lima. La aplicación de insecticida se llevó a cabo por: el Ministerio de Salud del Perú (MINSA), la Dirección General de Salud de las Personas (DGSP), la Estrategia Sanitaria Nacional de Prevención y Control de Enfermedades Metaxénicas y Otras Transmitidas por Vectores (ESNPCEMOTVS), la Dirección General de Salud Ambiental (DIGESA), el Gobierno Regional de Arequipa, la Gerencia Regional de Salud de Arequipa (GRSA), la Organización Panamericana de la Salud (OPS) y la Agencia Canadiense de Desarrollo Internacional (ACDI). También agradecemos a Michel Tibayrenc, Ben Bolker y un revisor anónimo.

References

1. WHO Expert Committee. (2002) Control of chagas disease. 109; WHO technical report series number 905: 1.
2. Levy MZ, Kawai V, Bowman NM, Waller LA, Cabrera L, et al. (2007) Targeted screening strategies to detect trypanosoma cruzi infection in children. PLoS Negl Trop Dis 1(3): e103.
3. Bowman NM, Kawai V, Levy MZ, Cornejo del Carpio JG, Cabrera L, et al. (2008) Chagas disease transmission in periurban communities of arequipa, peru. Clin Infect Dis 46(12): 1822-8.
4. Fraser B. (2008) Controlling chagas' disease in urban peru. Lancet 372(9632): 16-7.
5. Magalhaes JB, Pontes AL, Andrade SG. (1985) Behavior of the Y and peruvian strains of trypanosoma cruzi in mice, after passage through various media. Mem Inst Oswaldo Cruz 80(1): 41-50.
6. Lima ES, Andrade ZA, Andrade SG. (2001) TNF-alpha is expressed at sites of parasite and tissue destruction in the spleen of mice acutely infected with trypanosoma cruzi. Int J Exp Pathol 82(6): 327-36.
7. Schofield CJ, Jannin J, Salvatella R. (2006) The future of chagas disease control. Trends Parasitol 22(12): 583-8.
8. Pinto Dias JC. (1995) Natural history of chagas disease. Arq Bras Cardiol 65(4): 359-66.
9. Bern C, Montgomery SP, Herwaldt BL, A. R,Jr, Marin-Neto JA, et al. (2007) Evaluation and treatment of chagas disease in the united states: A systematic review. JAMA 298(18): 2171-81.
10. Maguire JH, Hoff R, Sherlock I, Guimaraes AC, Sleigh AC, et al. (1987) Cardiac morbidity and mortality due to chagas' disease: Prospective electrocardiographic study of a brazilian community. Circulation 75(6): 1140-5.
11. Reyes PA, Vallejo M. (2005) Trypanocidal drugs for late stage, symptomatic chagas disease (trypanosoma cruzi infection). Cochrane Database Syst Rev (4): CD004102.
12. Barbu C, Dumonteil E, Gourbiere S. (2009) Optimization of control strategies for non-domiciliated triatoma dimidiata, chagas disease vector in the yucatan peninsula, mexico. PLoS Negl Trop Dis 3(4): e416.
13. Zu Dohna H, Cecere MC, Gurtler RE, Kitron U, Cohen JE. (2009) Spatial re-establishment dynamics of local populations of vectors of chagas disease. PLoS Negl Trop Dis 3(7): e490.
14. Levy MZ, Bowman N, Kawai V, Waller LA, Cornejo del Carpio J, et al. (2006) Peri-urban infestation by triatoma infestans carrying trypanosoma cruzi in arequipa, peru. Emerging Infectious Diseases 12(9): 1345-1352.
15. Levy MZ, Kawai V, Bowman NM, Waller LA, Cabrera L, et al. (2007) Targeted screening strategies to detect trypanosoma cruzi infection in children. PLoS Negl Trop Dis 1(3): e103.
16. Mott KE, S. LJ,Jr, hoff R, Morrow RH, Muniz TM, et al. (1976) The epidemiology and household distribution of seroreactivity to trypanosoma cruzi in a rural community in northeast brazil. Am J Trop Med Hyg 25(4): 552-62.
17. Paz-Bailey G, Monroy C, Rodas A, Rosales R, Tabaru R, et al. (2002) Incidence of trypanosoma cruzi infection in two guatemalan communities. Trans R Soc Trop Med Hyg 96(1): 48-52.
18. Gurtler RE, Cecere MC, Lauricella MA, Petersen RM, Chuit R, et al. (2005) Incidence of trypanosoma cruzi infection among children following domestic reinfestation after insecticide spraying in rural northwestern argentina. Am J Trop Med Hyg 73(1): 95-103.
19. Black CL, Ocana S, Riner D, Costales JA, Lascanro MS, et al. (2007) Household risk factors for trypanosoma cruzi seropositivity in two geographic regions of ecuador. J Parasitol 93(1): 12-6.
20. Levy MZ, Bowman NM, Kawai V, Plotkin JB, Waller LA, et al. (2009) Spatial patterns in discordant diagnostic test results for chagas disease: Links to transmission hotspots. Clin Infect Dis 48(8): 1104-6.
21. Beale CM, Lennon JJ, Yearsley JM, Brewer MJ, Elston DA. (2010) Regression analysis of spatial data. Ecol Lett 13: 246–264.
22. Boyd HA, Flanders WD, Addiss DG, Waller LA. (2005) Residual spatial correlation between geographically referenced observations: A bayesian hierarchical modeling approach. Epidemiology 16(4): 532-41.
23. Okubo A, Levin SA, Okubo A. (2001) Diffusion and ecological problems : Modern perspectives. New York: Springer.
24. Smith DL, Lucey B, Waller LA, Childs JE, Real LA. (2002) Predicting the spatial dynamics of rabies epidemics on heterogeneous landscapes. Proc Natl Acad Sci U S A 99(6): 3668-72.
25. Keeling MJ, Woolhouse ME, Shaw DJ, Matthews L, Chase-Topping M, Haydon DT, Cornell SJ, Kappey J, Wilesmith J, Grenfell BT. (2001) Dynamics of the 2001 UK foot and mouth epidemic: Stochastic dispersal in a heterogeneous landscape. Science 26(5543): 813-817.
26. Jewell CP, Keeling MJ, Roberts GO. (2008) Predicting undetected infections during the 2007 foot-and-mouth disease outbreak. J R Soc Interface .
27. Legrand J, Egan JR, Hall IM, Cauchemez S, Leach S, et al. (2009) Estimating the location and spatial extent of a covert anthrax release. PLoS Comput Biol 5(1): e1000356.
28. Gelman A. (2004) Bayesian data analysis. Boca Raton, Fla.: Chapman & Hall/CRC.
29. Halloran ME. (1998) Concepts of infectious disease epidemiology. In: Rothman KJ, Greenland S, editors. Modern Epidemiology. Philadelphia, PA: Lippincott-Raven. pp. 529-554.

30. Muench H. (1959) Catalytic models in epidemiology. Cambridge,: Harvard University Press. 110 p. p.
31. Gurtler RE, Cecere MC, Lauricella MA, Cardinal MV, Kitron U, et al. (2007) Domestic dogs and cats as sources of trypanosoma cruzi infection in rural northwestern argentina. Parasitology 134(Pt 1): 69-82.
32. McCullagh P, Nelder JA. (1998) Generalized linear models. Boca Raton: Chapman & Hall/CRC. 511 p. p.
33. Gilks WR, Richardson S, Spiegelhalter DJ. (1998) Markov chain monte carlo in practice. Boca Raton, Fla.: Chapman & Hall.
34. Kass RE, Raftery AE. (1995) Bayes factors. Journal of the American Statistical Association 90: 773-795.
35. Geweke J. (1992) Evaluating the accuracy of sampling-based approaches to calculating posterior moments. In: Bernado JM, Berger JO, Dawid AP, Smith AFM, editors. Bayesian Statistics 4. Oxford, UK: Clarendon Press.
36. Gelman A, Rubin Donald B. (1992) Inference from iterative simulation using multiple sequences. Statistical Science 7(4): 457-472.
37. Breniere SF, Llanos B, Tibayrenc M, Desjeux P. (1985) Isoenzymic studies and epidemiological data of trypanosoma cruzi from arequipa (peru), pacific side. Ann Soc Belg Med Trop 65 Suppl 1: 63-6.
38. Breniere SF, Lopez J, Vargas F, Barnabe C. (1997) Genetic variability and microdistribution of triatoma infestans genotypes and trypanosoma cruzi clones in arequipa region (peru). Mem Inst Oswaldo Cruz 92(3): 401-8.
39. Brisse S, Verhoef J, Tibayrenc M. (2001) Characterisation of large and small subunit rRNA and mini-exon genes further supports the distinction of six trypanosoma cruzi lineages. Int J Parasitol 31(11): 1218-1226.
40. Viotti R, Vigliano C, Lococo B, Bertocchi G, Petti M, et al. (2006) Long-term cardiac outcomes of treating chronic chagas disease with benznidazole versus no treatment: A nonrandomized trial. Ann Intern Med 144(10): 724-34.
41. Sosa-Estani S, Segura EL. (2006) Etiological treatment in patients infected by trypanosoma cruzi: Experiences in argentina. Curr Opin Infect Dis 19(6): 583-7.
42. Cohen JE, Gurtler RE. (2001) Modeling household transmission of american trypanosomiasis. Science 293(5530): 694-8.
43. Bayer AM, Hunter GC, Gilman RH, Cornejo Del Carpio JG, Naquira C, et al. (2009) Chagas disease, migration and community settlement patterns in arequipa, peru. PLoS Negl Trop Dis 3(12): e567.
44. Melbourne BA, Hastings A. (2009) Highly variable spread rates in replicated biological invasions: Fundamental limits to predictability. Science 325(5947): 1536-9.
45. Green PJ. (1995) Reversible jump markov chain monte carlo computation and bayesian model determination. Biometrika 82: 711-732.
46. Delgado S, Castillo Neyra R, Quispe Machaca VR, Ancca Juárez J, Chou Chu L, Verastegui MR, Moscoso Apaza GM, Bocángel CD, Tustin AW, Sterling CR, Comrie AC, Náquira C, Cornejo del Carpio JG, Gilman RH, Bern C, Levy MZ. (2011) A history of chagas disease transmission, control, and re-emergence in peri-rural La Joya, Peru. PLoS Negl Trop Dis 5(2): e970.

LEYENDA DE FIGURAS COMPLEMENTARIA

Texto S1 Evidencia de ausencia de la fase tardía de la enfermedad de Chagas.

Texto S2 Descripción de variables de regresión para epicentro en vídeo. Un compendio visual de las animaciones para describir el proceso de adaptación del método Monte Carlo con cadenas de Markov al *epicenter regression*.

Video S1- Animación de un epicentro. Modelo de un epicentro: animación que describe el proceso de adaptación del método Monte Carlo con cadenas de Markov al *epicenter regression* para los datos de infestación de *T. cruzi* en Arequipa, Perú.

Video S2- Animación de dos epicentros. Modelo de dos epicentros: animación que describe el proceso de adaptación del método Monte Carlo con cadenas de Markov al *epicenter regression* para los datos de infestación de *T. cruzi* en Arequipa, Perú.

Video S3- Animación de tres epicentros. Modelo de tres epicentros: animación que describe el proceso de adaptación del método Monte Carlo con cadenas de Markov al *epicenter regression* para los datos de infestación de *T. cruzi* en Arequipa, Perú.

Video S4- Animación de cuatro epicentros. Modelo de cuatro epicentros: animación que describe el proceso de adaptación del método Monte Carlo con cadenas de Markov al *epicenter regression* para los datos de infestación de *T. cruzi* en Arequipa, Perú.

Texto S3 Léame, Explicación del Código. El archivo 'Léame' describe los códigos que acompañan al *epicenter regression*. Conjunto de datos S1, datos simulados para *epicenter regression*. Datos simulados de un modelo Susceptible de Infección (SI) en una tabla que debe utilizarse cuando se ejecuta *epicenter regression* y los códigos de la película.

Texto S4 Código del modelo para *epicenter regression*. Código de *epicenter regression* (en R). El código contiene notas para describir el proceso de adaptación del método Monte Carlo con cadenas de Markov.

Texto S5 Código de la película de *epicenter regression*. Código R utilizado para producir películas similares a las de los materiales complementarios del manuscrito.
