# Supplementary material for: Retracing Micro-Epidemics of Chagas Disease Using Epicenter Regression
Source: PLoS Comput Biol. 2011 Sep 15;7(9):e1002146. doi: 10.1371/journal.pcbi.1002146 (PMC3174153; doi:10.1371/journal.pcbi.1002146)
Supplement: Text S1 — Evidence of Lack of Late Stage Chagas Disease. (DOCX) [file pcbi.1002146.s003.docx]

**Supplemental materials**

**S1 text. Evidence of lack of late chronic stage cardiac Chagas disease in hospitals in Arequipa, Peru.**

In order to verify the anecdotal absence of late-stage Chagas disease in hospitals in Arequipa, Peru, free screening for *T. cruzi* infection was offered to patients seeking care in the cardiology department of the Honorio Delgado hospital between March and December 2008. Honorio Delgado is one of two large public hospitals in the city of Arequipa and the referral center for Chagas disease for the greater metropolitan area of the city. The hospital’s large catchment area includes Guadalupe and many similar peri-urban communities. Conventional serological testing was conducted using a commercial ELISA and IFA following published methods. Persons with *T. cruzi* infection confirmed by both positive serological tests underwent a history and physical examination, electrocardiogram and echocardiogram. The hospital-based study was approved by the Institutional review board of the Johns Hopkins University and Asociacion Benefica Prisma.

The cardiac form of Chagas disease is often classified by severity (39, 40). One commonly used classification system (Acquatella) identifies progressively more severe stages (40). The first (A) is equivalent to the indeterminate form of Chagas disease. It is characterized by the absence of signs or symptoms, though affected individuals may manifest non-specific electrocardiogram (ECG) abnormalities (39). Mild symptoms may occur in the second stage (B), which is characterized by more advanced and specific conduction abnormalities, and carries a risk of sudden cardiac death. The third (C) and fourth (D) stages are fully symptomatic; there is continued risk of sudden cardiac death and moderate to severe congestive heart failure (39, 40)

Only fifteen of 423 (3.54%) patients who presented to the cardiology ward in the Honorio Delgado hospital in Arequipa were confirmed seropositive for *T. cruzi* infection. Based on physical examination, ECG and echocardiogram data, each patient received a Chagas cardiomyopathy severity classification. Of the 15 confirmed seropositive individuals, none was diagnosed with late stage (stage C or D) Chagas disease. By contrast, in a parallel study in the cardiology ward of the Hospital Universitario Japones, in the city of Santa Cruz, Bolivia, where Chagas transmission is endemic, 60.5% (189/312) of outpatients tested between August and November of 2008 were infected with *T. cruzi*, and 33/150 (22.0%) patients evaluated had late stage (C or D) Chagas disease.
