# Supplementary material for: Retracing Micro-Epidemics of Chagas Disease Using Epicenter Regression
Source: PLoS Comput Biol. 2011 Sep 15;7(9):e1002146. doi: 10.1371/journal.pcbi.1002146 (PMC3174153; doi:10.1371/journal.pcbi.1002146)

Movie of fitting Guadalupe data with 3 epicenters

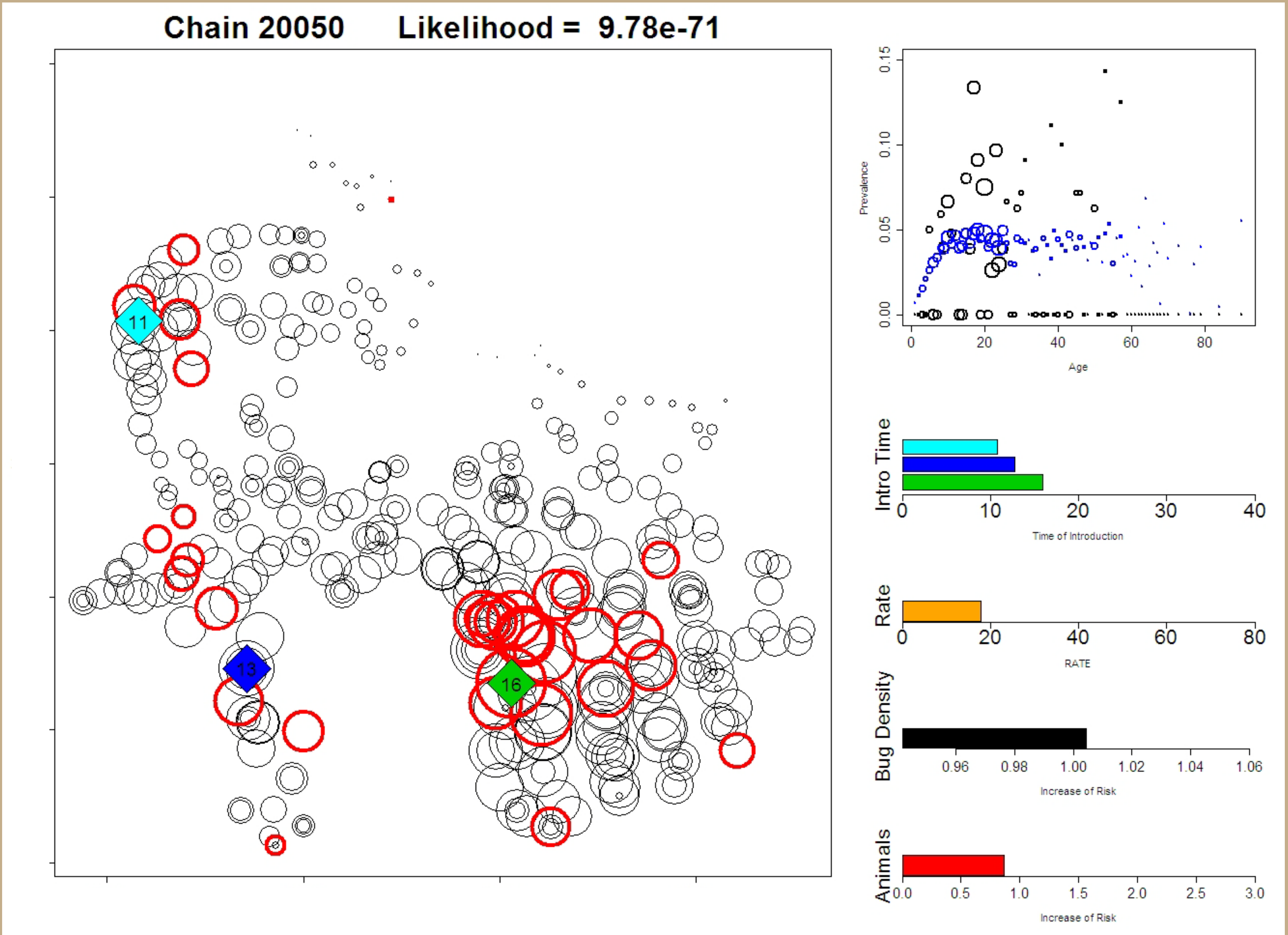

Movie of fitting Guadalupe data with 3 epicenters

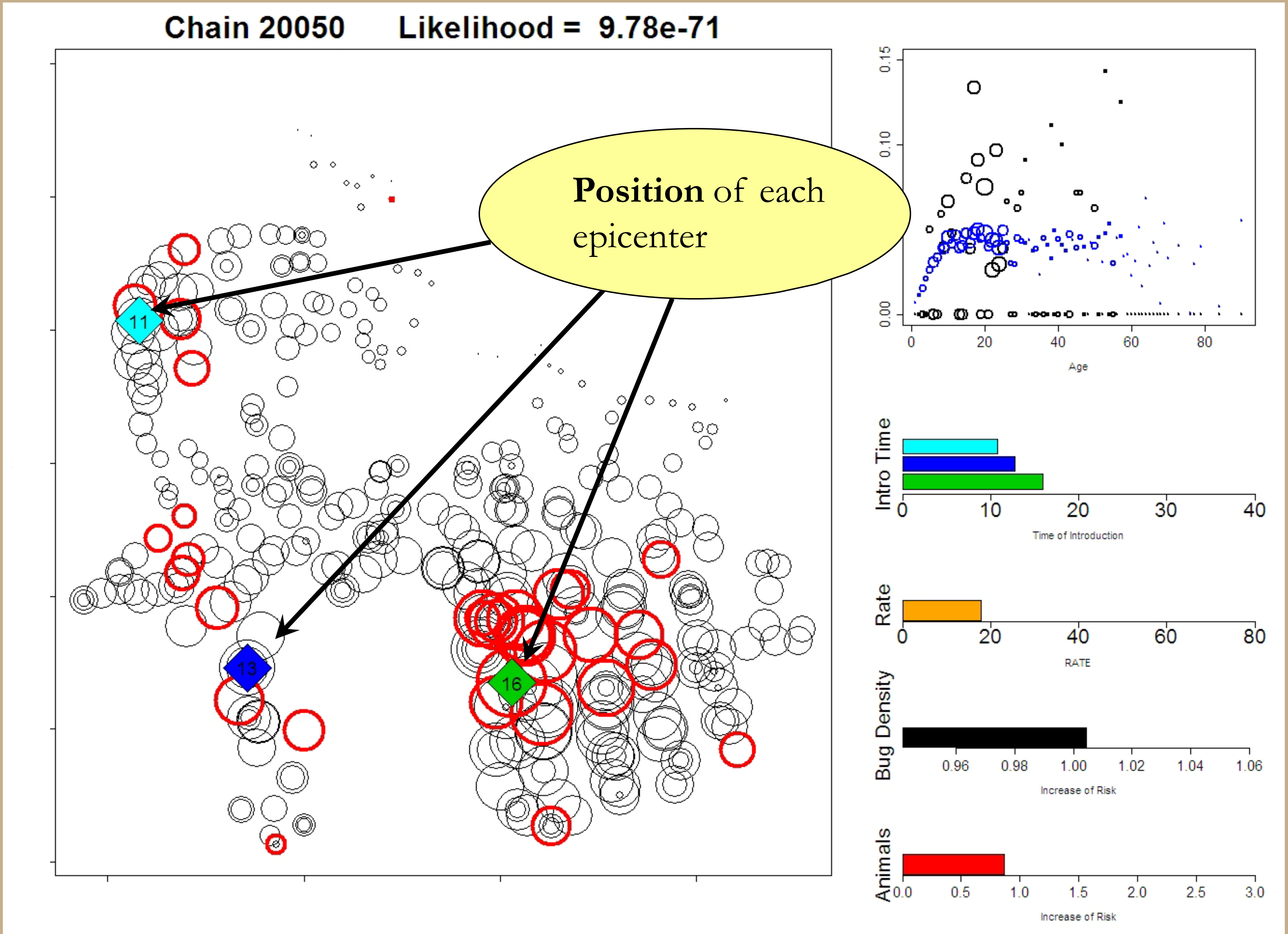

Movie of fitting Guadalupe data with 3 epicenters

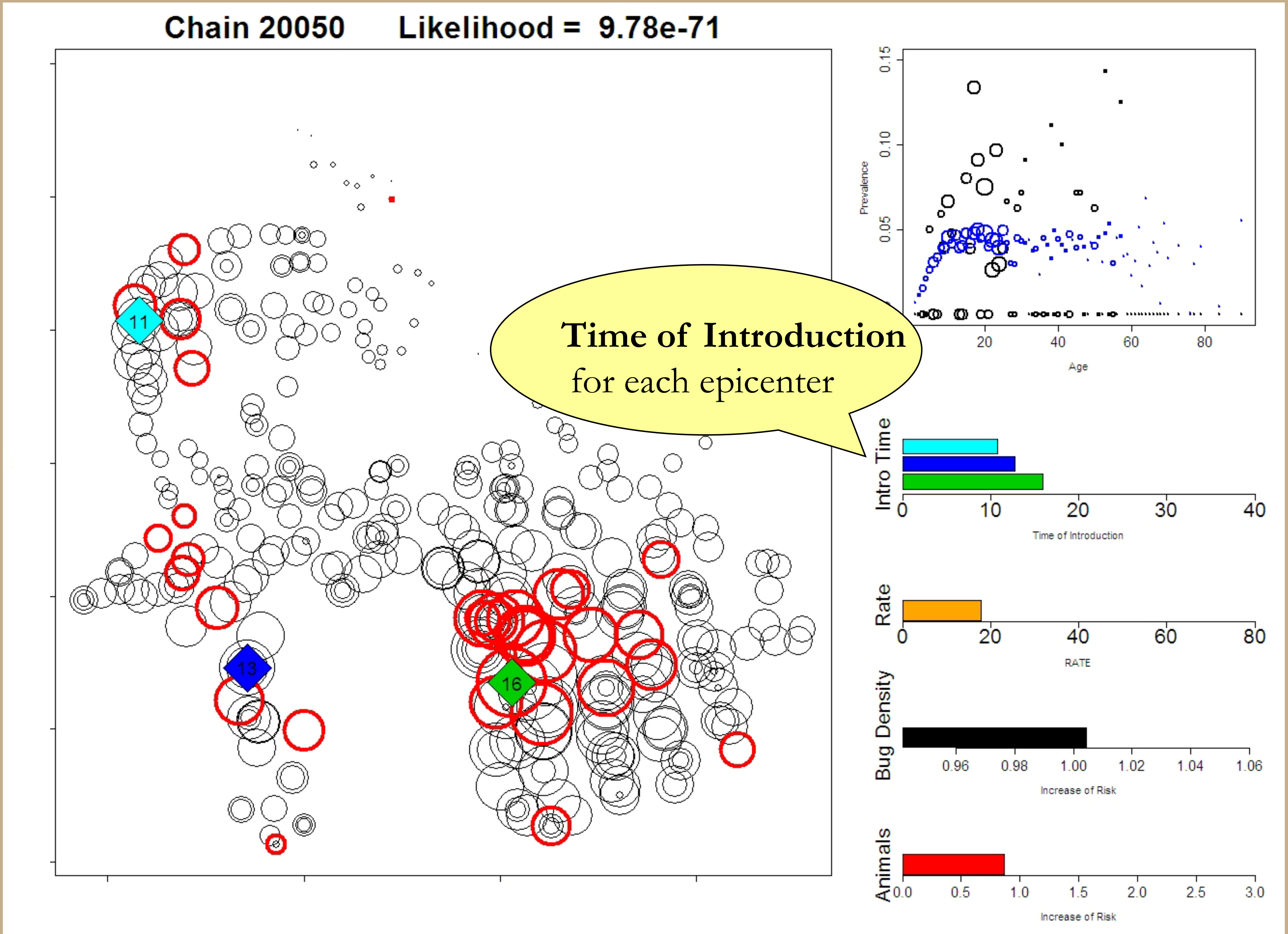

Movie of fitting Guadalupe data with 3 epicenters

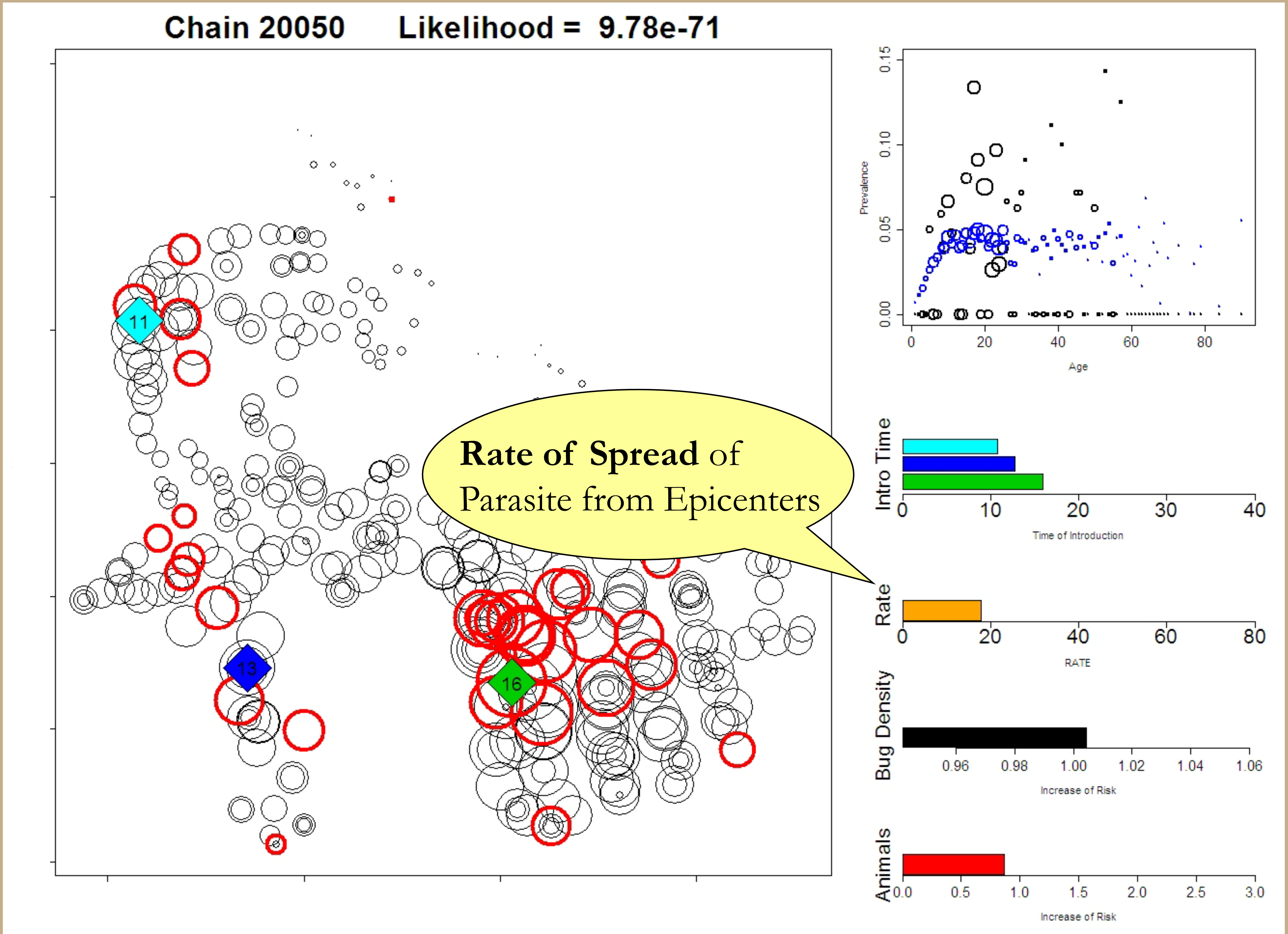

Movie of fitting Guadalupe data with 3 epicenters

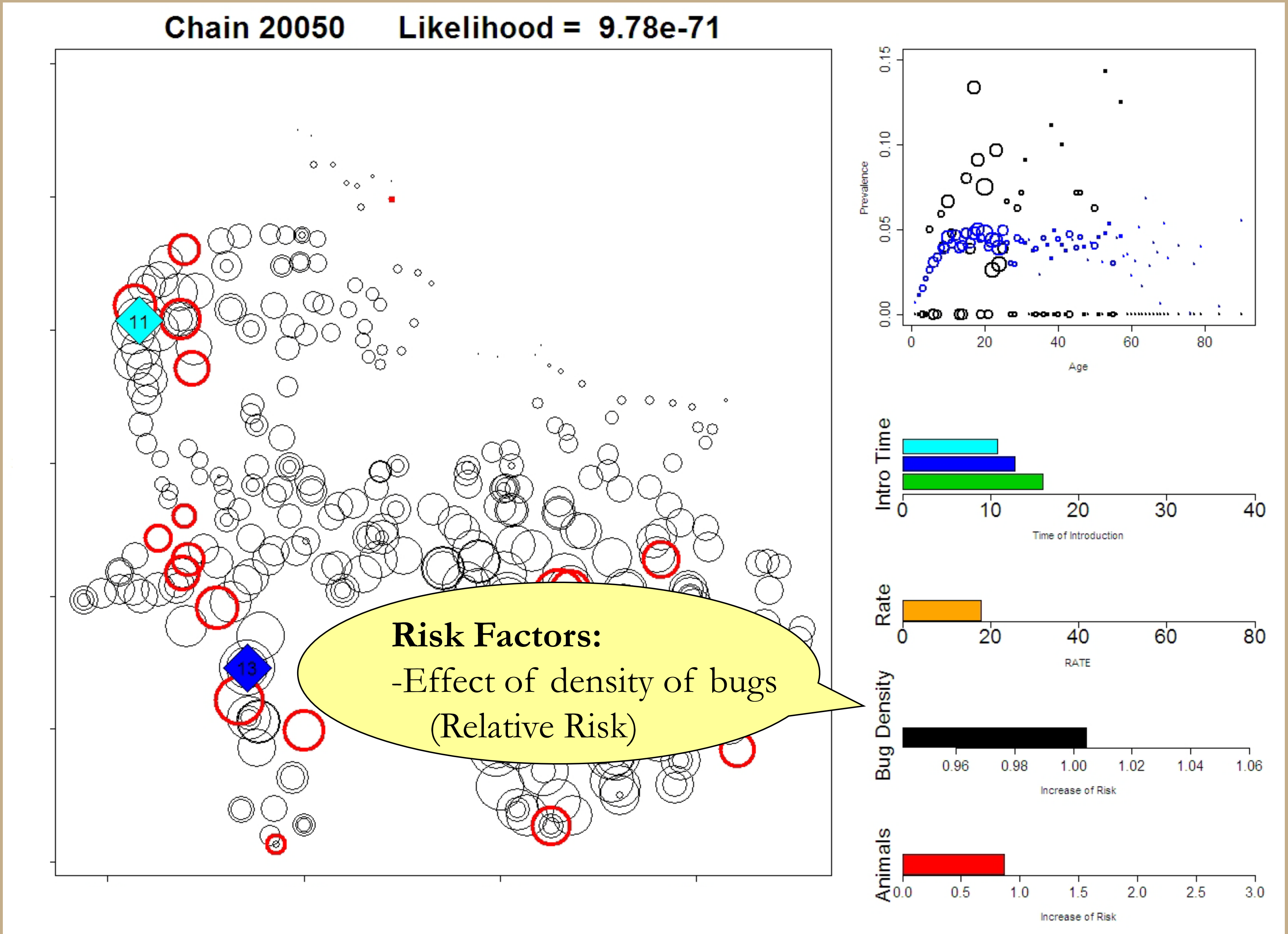

Movie of fitting Guadalupe data with 3 epicenters

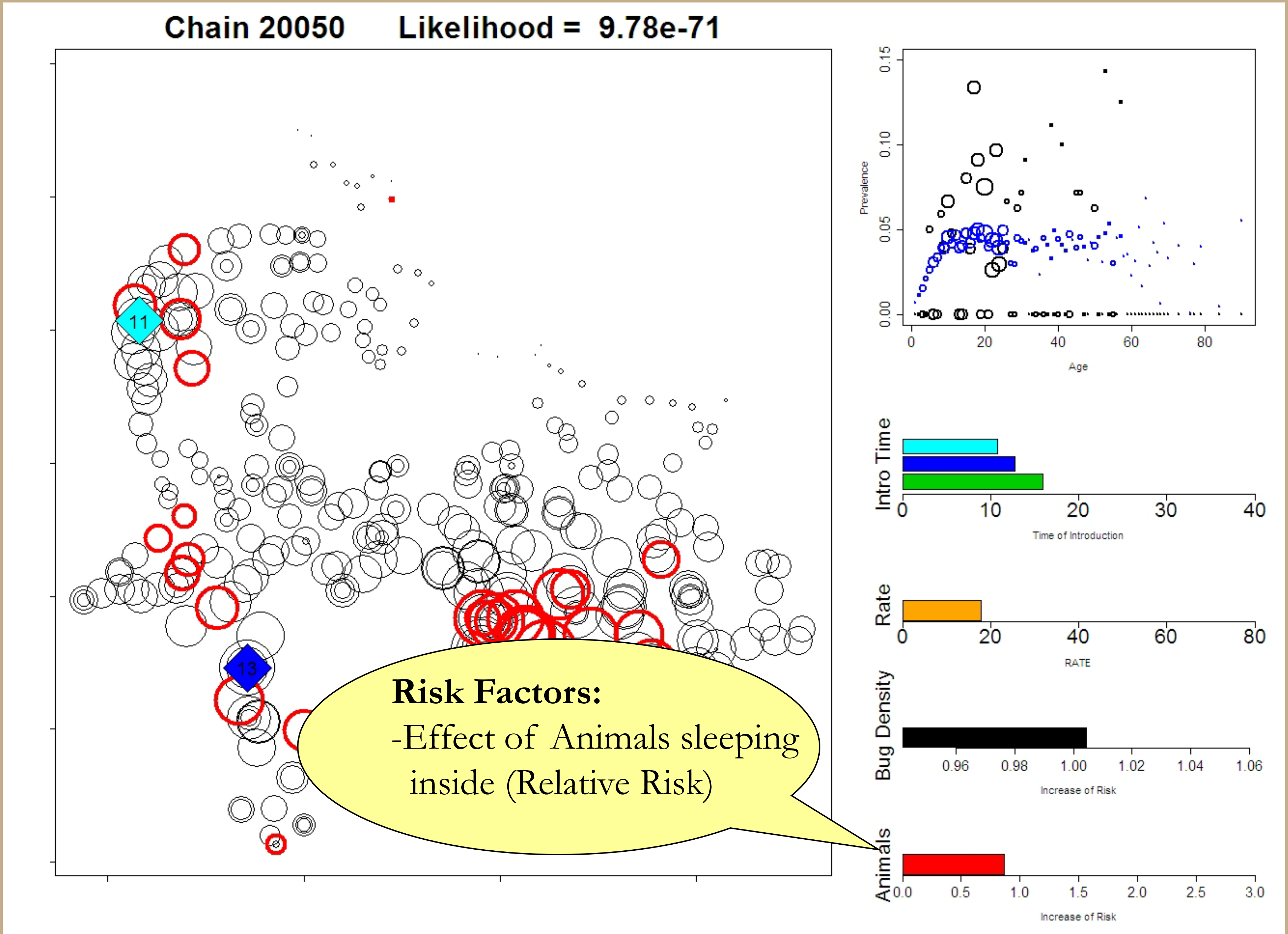

Movie of fitting Guadalupe data with 3 epicenters

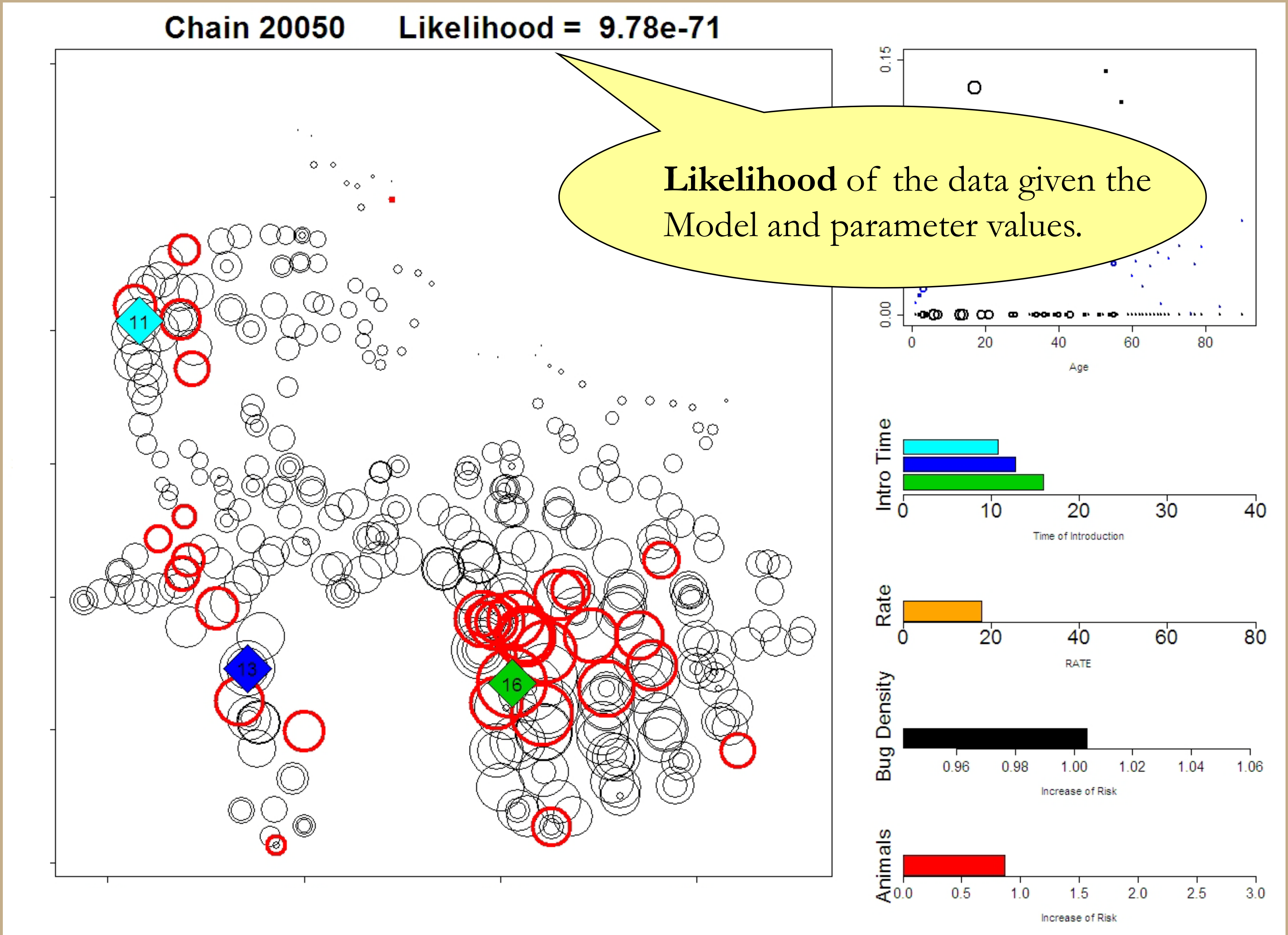

Movie of fitting Guadalupe data with 3 epicenters

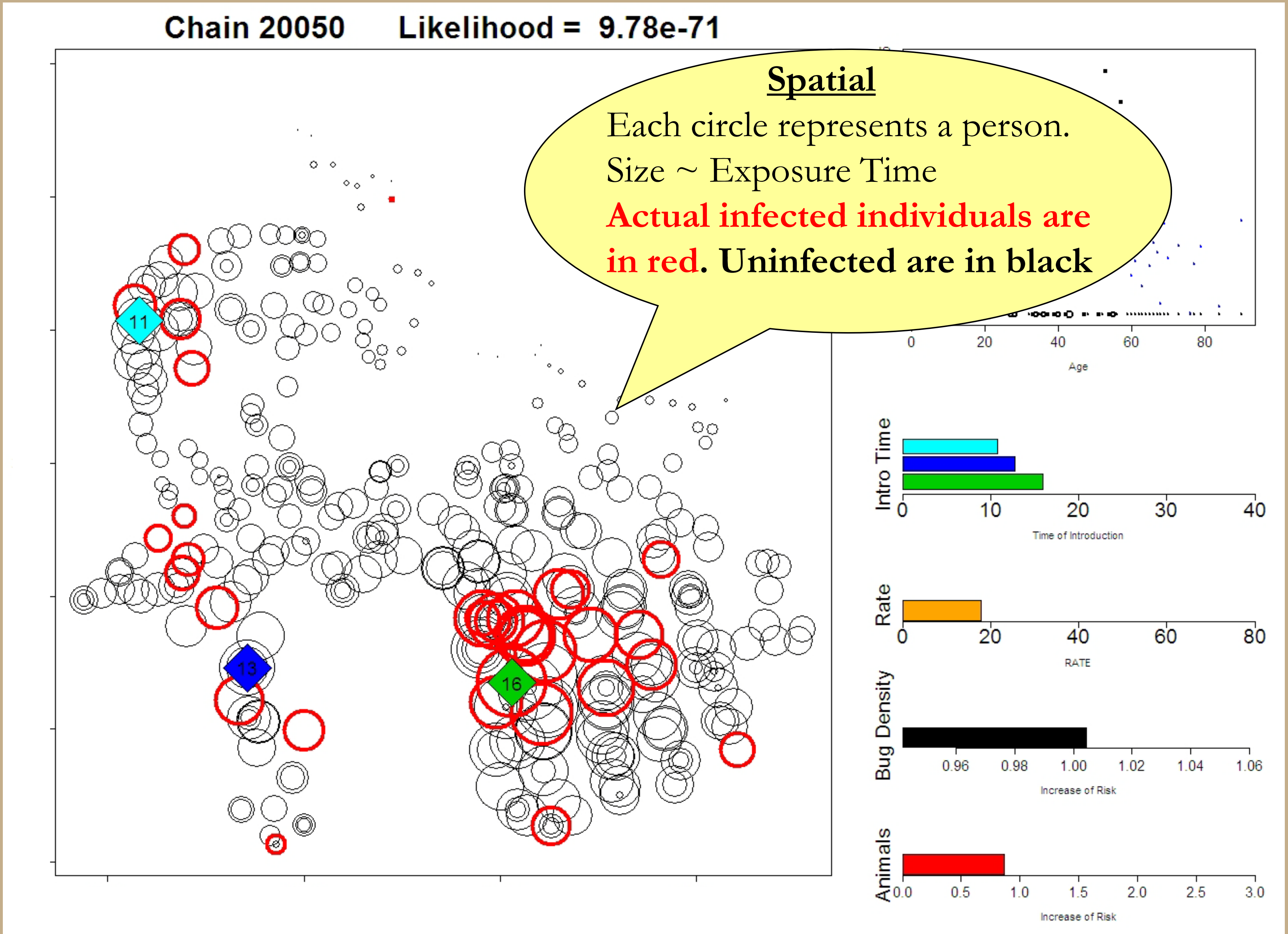

Movie of fitting Guadalupe data with 3 epicenters

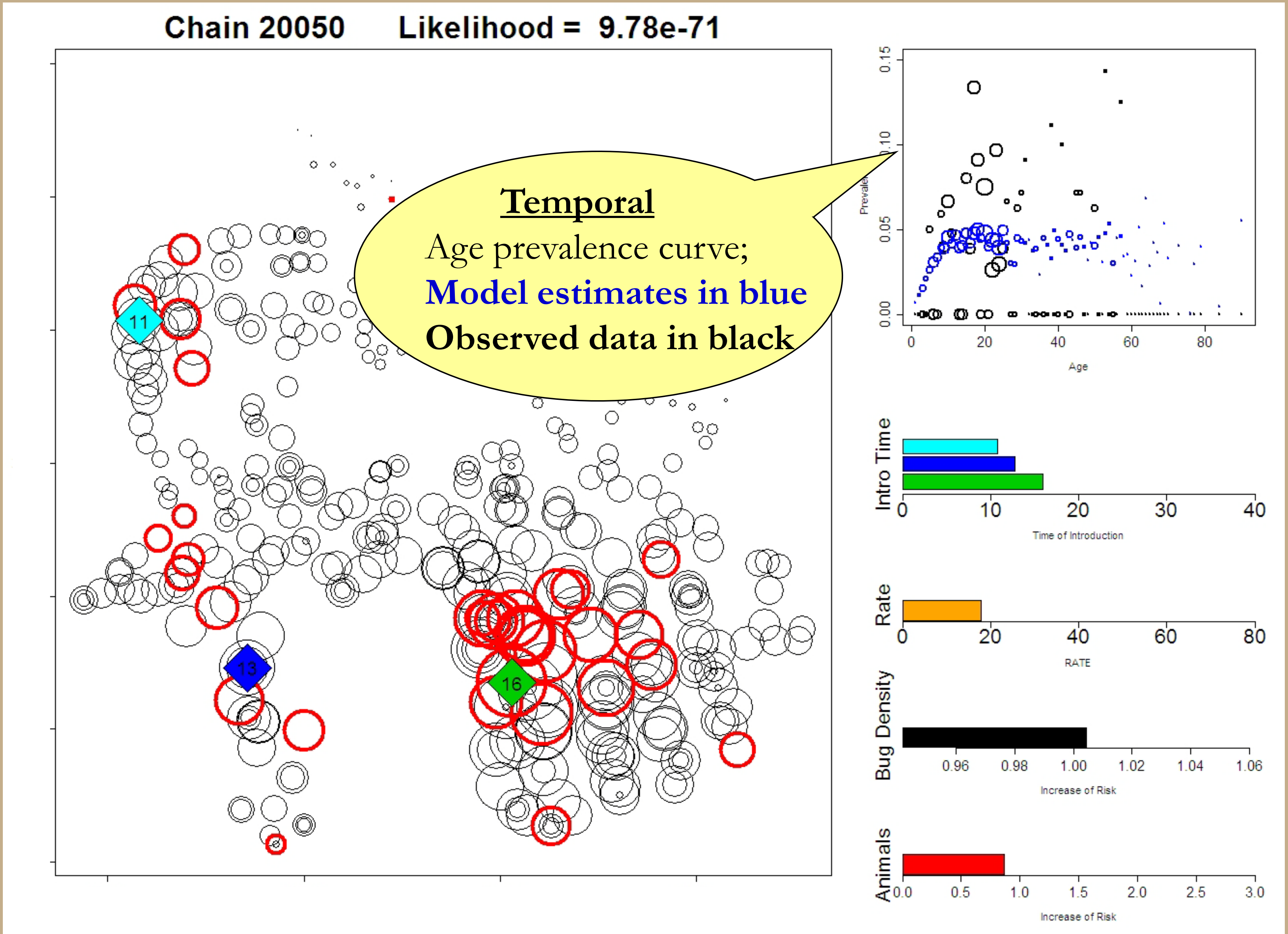

Supplement: Text S2 — Description of epicenter regression variables in video. A visual compendium to the animations of describing the fitting process of the Monte Carlo Markov Chains for epicenter regression. (PDF) [file pcbi.1002146.s004.pdf]
