# Supplementary material for: Retracing Micro-Epidemics of Chagas Disease Using Epicenter Regression
Source: PLoS Comput Biol. 2011 Sep 15;7(9):e1002146. doi: 10.1371/journal.pcbi.1002146 (PMC3174153; doi:10.1371/journal.pcbi.1002146)
Supplement: Text S3 — Read Me Code Explanation. A ‘Readme’ file describing the accompanying epicenter regression codes. (DOCX) [file pcbi.1002146.s005.docx]

*Retracing micro-epidemics of Chagas disease using epicenter regression*

This zip file includes:

1. A sample dataset, Dataset S1, SimulatedData.csv. This dataset is a Susceptible Infectious (SI) model simulated on a grid.

2. The Epicenter regression code used, Text S4, Epicenter_Regression_Model_Simulated_Data.r. The code is annotated throughout especially for the Monte Carlo Markov Chain methods. At the top of the code are simulation parameters, including the number of epicenters, the number of links in the chain to be produced and the amount of thinning of the chains that is to be done. Initial parameters can be changed to accommodate other models. Currently, the code will import the simulated data into your R workspace. It is set to produce an output called ‘CHAINS1’. CHAINS1 is an MCMC chain, 100 lines long after being thinned by a factor of 10.

3. Text S5, Epicenter_Regression_Movie_Code_Simulated_Data.r, is the code used to produce movies similar to those in the supplemental materials of the manuscript. These movies visualize the fitting process of epicenter regression to Chagas disease data from Arequipa, Peru. The code here produces similar movies on the epicenter regression predictions for the sample dataset. Each frame of the movie represents a single link in the MCMC chain. The points in the main figure are scaled in size based on the their time of exposure. Cases are shown in red. Uninfected individuals are in black. The figure on the top right shows the age prevalence curve with model estimates in blue and observed data in black. The first and second bar graphs show the times of introduction of the first and second epicenter, respectively. The third bar graph shows the proposed rate of spread of the disease agent. The final bar graph shows the effect of a risk factor that affects the probability of infection given exposure.

**How to run the epicenter regression code**

1. Open R and set your working directory to the location where you saved the epicenter regression code and data files. The command in R to set your working directory is setwd(“example/directory”).

2. Open Text S4, Epicenter_Regression_Model_Simulated_Data.r. This will likely open into a text window of R. Copy and paste everything in this text window into your R workspace. This will import the datafile Simulated_data.csv, run the epicenter regression model, and produce and MCMC chain called ‘CHAINS1’.

3. Open Text S5, Epicenter_Regression_Model_Simulated_Data.r. Again, this will likely open in a text window of R. Copy and paste from the first line of this code through the last line of the MOVIE function.

4. There are alternative codes to open a graphics window for Mac and Windows systems (see annotations in code).

5. Copy and paste the next line of the Text S5, Epicenter_Regression_Movie_Code_Simulated_Data.r. It will be the line to run the MOVIE function. It will look like MOVIE(chains_data, 2, 1,100).

6. In your graphics window you will see the visualization of the first line of the chain. Click on your R workspace and hit “return” to visualize the next line in the chain.

7. Hit escape to finish.
